# Improved Interfacial Electron Dynamics with Block Poly(4-vinylpyridine)–Poly(styrene) Polymers for Efficient and Long-Lasting Dye-Sensitized Solar Cells

**DOI:** 10.1021/acsapm.4c01238

**Published:** 2024-07-20

**Authors:** Daniela F. S. L. Rodrigues, Carlos M. R. Abreu, Frédéric Sauvage, Jorge F. J. Coelho, Arménio C. Serra, Dzmitry Ivanou, Adélio Mendes

**Affiliations:** † CEMMPRE, ARISE, Department of Chemical Engineering, University of Coimbra, Rua Sílvio Lima—Polo II, 3030-790 Coimbra, Portugal; ‡ LEPABE, Departamento de Engenharia Química, Faculdade de Engenharia, Universidade do Porto, Rua Dr. Roberto Frias, 4200-465 Porto, Portugal; § Laboratoire de Réactivité et Chimie des Solides, Université de Picardie Jules Verne (UPJV), CNRS UMR 7314, Hub de l’énergie, 15 rue Baudelocque, 80039 Amiens, France; ∥ IPN, Instituto Pedro Nunes, Associação para a Inovação e Desenvolvimento em Ciência e Tecnologia, Rua Pedro Nunes, 3030-199 Coimbra, Portugal; ⊥ ALiCE—Associate Laboratory in Chemical Engineering, Faculty of Engineering, University of Porto, Rua Dr. Roberto Frias, Porto 4200-465, Portugal

**Keywords:** indoor and outdoor photovoltaics, thin-film solar cell, interfacial electron dynamics, coadsorbents, stability

## Abstract

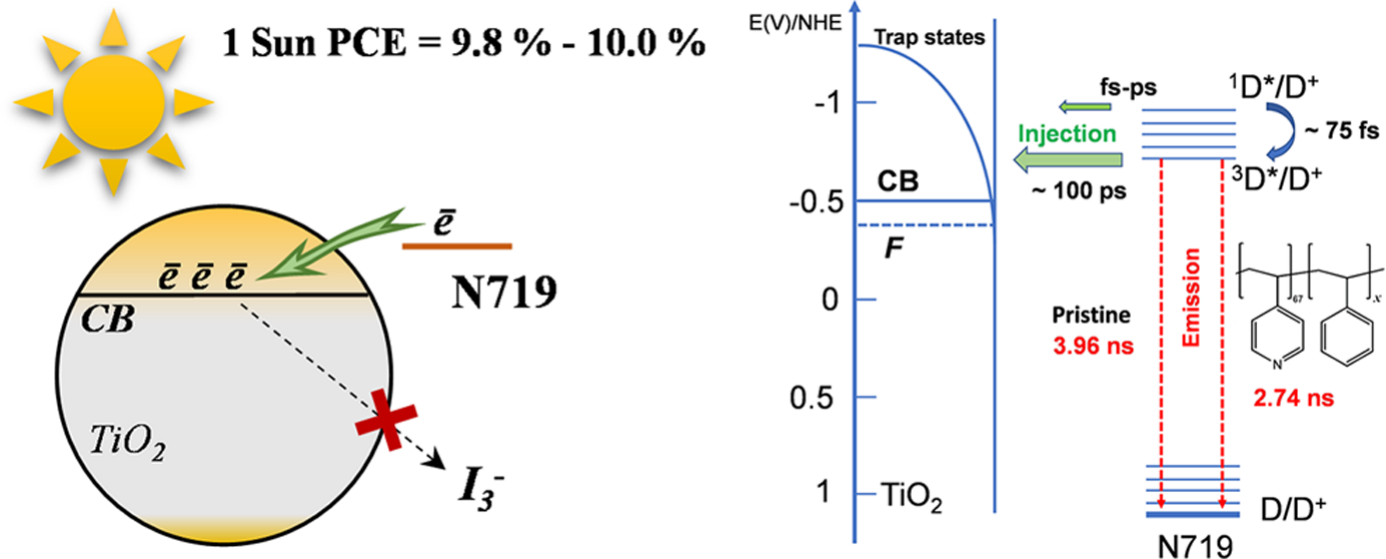

Dye-sensitized solar cells (DSSCs) have recently entered the market for indoor photovoltaics. Fast electron injection from dye to titania, the lifetime of the excited dye, and the suppression of back electron recombination at the photoanode/electrolyte interface are crucial for a high photocurrent conversion efficiency (PCE). This study presents block copolymers of poly(4-vinylpyridine) and poly(styrene)-P4VP_67_-*b*-PSt_*x* (*x*=23;61)_ as efficient accelerators of electron injection from dye to titania with extended lifetime excited states and long-lasting back electron recombination suppression. P4VP_67_-*b*-PSt_23_ and P4VP_67_-*b*-PSt_61_ rendered devices with PCEs of 10.0 and 9.8%, respectively, under AM 1.5G light; PCEs of 19.4 and 16.4% under 1000 lx LED light were attained. Copolymers provided a stable PCE with the two most popular I_3_^–^/3I^–^ electrolytes based on ACN and 3-methoxypropionitrile solvents; PCE history was tracked in the dark and under 1000 h of continuous light soaking with passive load according to ISOS-D1 and ISOS-L2 aging protocols, respectively. The impact of the polymer molecular structure on electron recombination, charge injection, dye anchoring, light absorption, photocurrent generation, and PCE and the long-term history of photovoltaic metrics are discussed.

## 1 Introduction

Over the last three decades, dye-sensitized solar cells (DSSCs) have gained prominence in scientific and technological circles due to their straightforward manufacturing, high photocurrent conversion efficiency (PCE) under low and diffuse lighting, and ability to be crafted into flexible, semitransparent modules that are both aesthetically pleasing and offer innovation potential.^1−3^ Independent research facilities confirmed a PCE of 15.2%.^4,5^

A superior PCE in low-light conditions and the capability to customize the transparency of modules make DSSCs ideal for photovoltaic applications in building glazing, greenhouses, and agricultural systems.^6−8^ Emerging applications cover electricity storage,^9^ solar-powered flow batteries,^10^ self-powered water-splitting systems,^11,12^ autonomous clinical diagnostic biosensors,^13^ and wearable technologies,^14,15^ highlighting the versatility of DSSC technology. The increasing need for off-grid electricity solutions for low-power electronics and the Internet of Things (IoT) amplifies the interest in DSSCs, renowned for their efficient indoor light-to-electricity conversion,^16−19^ with possible PCEs around 40% from artificial indoor lighting,^19^ and reported up to 35%.^4,20^ The recent market entry of DSSCs by various companies for indoor photovoltaic (PV) systems highlights their increasing commercial importance.^1,16^ This underlines the critical need to develop affordable, high-performing materials for stable and efficient devices.

In enhancing the PCE, coadsorbents at the photoanode/electrolyte interface are vital. These amphiphilic molecules often feature a hydrophobic core with functional groups like carboxylic or phosphonic acids that anchor to mesoporous structures, blocking gaps, preventing dye aggregation, and suppressing back electron transfer.^21−25^ Chenodeoxycholic acid (CDCA), introduced to DSSCs in 1993,^26^ remains a standard for high-efficiency, stable devices. CDCA favors dye dispersion and TiO_2_ coverage, reduces recombination, and promotes charge separation.^27−30^ Despite its effectiveness, the production of CDCA, typically extracted from animal livers, is not only costly but also ethically concerning.^31^

Efforts to discover synthetic alternatives to CDCA have led to exploring various small molecular coadsorbents and additives.^22,32−34^ However, achieving both a high PCE and long-term stability remains challenging. Dendritic macromolecules and polymers have shown promise, achieving PCEs of about 7.8, 5.8, and 5.7%^34−36^ though they often underperform compared to CDCA-treated cells.^37^ A notable synthetic coadsorbent is poly(4-vinylpyridine) (P4VP), which allowed PCEs of 9% under AM 1.5G illumination and 22% under artificial lighting.^38^ However, its limited stability in DSSCs due to poor sorption strength and desorption has been problematic, leading to PCE degradation.

This work presents block copolymers P4VP_67_-*b*-PSt_*x* (*x*=23;61)_, which improve interfacial electron dynamics on the titania/dye interface and electron injection from the dye to TiO_2_, prolong dye excited states, and possess efficient back electron recombination suppression. Stability issues are solved by the molecular engineering of polymer chains that are insoluble in the most used DSSC electrolytes. P4VP_67_-*b*-PSt_*x* (*x*=23;61)_ enabled the fabrication of devices with high 1 sun PCEs of ca. 9 and 6% with ACN and 3-methoxypropionitrile electrolytes, respectively; devices with polymeric coadsorbents retained their PCEs after 1000 h of an accelerated ISOS-L2 light soaking test. This makes fully synthetic P4VP_67_-*b*-PSt_*x* (*x*=23;61)_ attractive for efficient and durable DSSCs for solar and artificial light conversion devices.

## 2 Materials and Methods

### 2.1 Synthesis of P4VP and P4VP-*b*-PSt Block Copolymers

**Figure 1:**
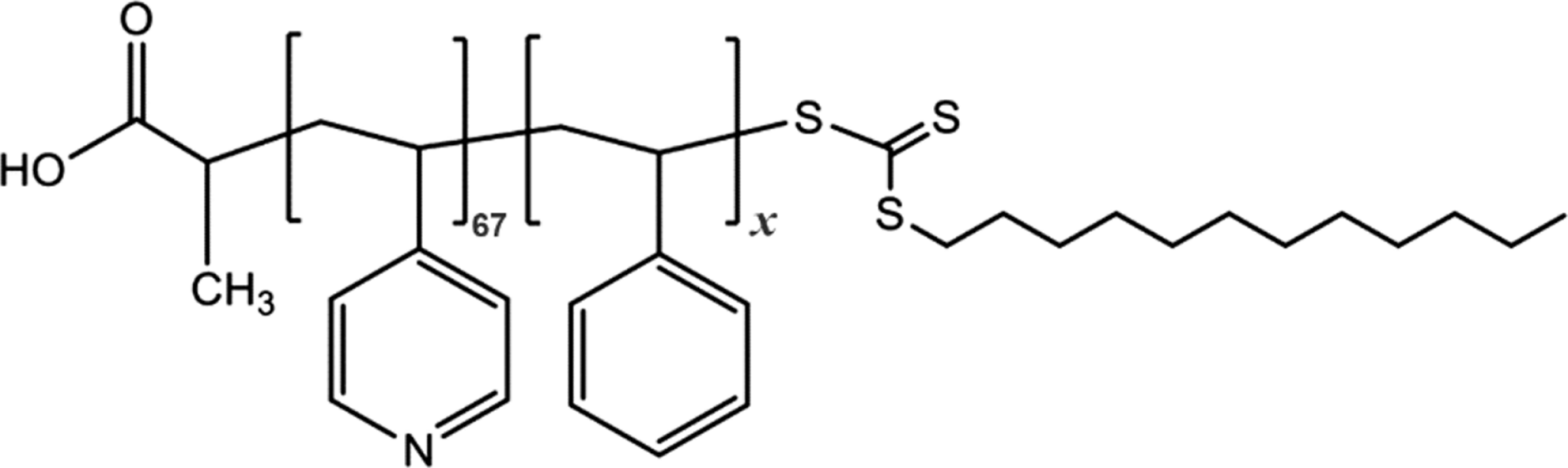
Molecular structure of P4VP_67_-*b*-PSt_*x* (*x*=23;61)_ block copolymers.

The synthesis and characterization of P4VP are described in our previous work^38^ using RAFT polymerization.^39,40^ P4VP used in this study has 77 repetition units of 4-vinilpyridine and a molecular weight (*M*_W_) of 8.5 kg·mol^–1^. Two block copolymers of poly(4-vinylpyridine)-*b*-poly(styrene) (P4VP-*b*-PSt) with *M*_W_ of 9.8 and 13.7 kg mol^–1^ were produced by RAFT polymerization (Scheme S1, Supporting Information). ^1^H NMR spectroscopy (Figure S1) confirmed the structure of the copolymers. The copolymers were obtained with a low dispersity (*Đ*) (≤1.3) (Table S1); the *M*_W_ distribution curves obtained by SEC are presented in Figure S2. The copolymers are prepared from a P4VP block and a PSt block, as shown in Figure 1. Both copolymers have the same length of the P4VP chain (67 units of 4-vinylpyridine) and different lengths of the PSt chain. Polymers with 23 and 61 St units are denoted as P4VP_67_-*b*-PSt_23_ and P4VP_67_-*b*-PSt_61_, respectively.

### 2.2 DSSC Fabrication

The general assembling procedures of DSSC devices, materials, and reagents are described elsewhere.^37,38^ Briefly, to produce counter electrodes, fluorine-doped tin oxide (FTO)-coated glasses (TEC-7; 7 Ω/sq; 1.5 × 1.5 cm^2^) were predrilled with two holes for electrolyte injection, cleaned, and activated with Pt nanoparticles by thermolysis at 500 °C for 1 h of the Platisol T/SP (Solaronix) paste. For the preparation of photoanodes, first a blocking layer (80 ± 5 nm) of dense TiO_2_ was deposited on FTO-coated glass by spray pyrolysis. A circular-shaped (0.13 cm^2^) two-layered mesoporous titania scaffold was deposited on top of the TiO_2_ blocking layer; DN-EP02 (18–20 nm anatase, Dyenamo) and 18NR-AO (GreatCell Solar) TiO_2_ pastes were sequentially screen-printed to obtain transparent (8.3 μm) and light-scattering (5.7 μm) TiO_2_ layers, respectively (Figure S3). Screen-printed pastes were dried at 120 °C for 15 min and then sintered at 500 °C for 1 h. The glass substrates with a mesoporous TiO_2_ scaffold were subjected to treatment in an aqueous solution of TiCl_4_ (20 min, 70 °C) followed by sintering at 500 °C for 1 h.

Sensitization of TiO_2_ was performed by sequential adsorption of the N719 dye and P4VP_67_-*b*-PSt_*x* (*x*=23;61)_ polymers at ca. 20 ± 1 °C. First, the dye was adsorbed on TiO_2_ by dipping in 0.3 mM N719 solution in absolute ethanol for 24 h. Then, photoanodes were rinsed with ethanol, immersed in 30 μM polymer solution in ethanol for 12 ± 2 h, rinsed with ethanol, and dried under a nitrogen flow. Sequential adsorption of the dye and polymer leads to better-performing devices compared to their counterparts obtained by simultaneous adsorption.^38^ Polymers compete with the dye in the adsorption process, resulting in less dye being adsorbed during cosensitization, especially at high polymer concentrations. In the sequential method, the amount of the preadsorbed dye remains ∼0.15 ± 0.01 mg·cm^–2^ regardless of the polymer concentration. Interested readers can consult the previous study^38^ where the impact of coadsorbent-loading procedures (sequential and simultaneous) on dye loading and photogeneration/recombination was studied in detail over a wide concentration range of P4VP with different *M*_W_. These findings were explained through the analysis of TiO_2_–polymer, TiO_2_–dye, and TiO_2_/dye–polymer interactions. Photoanodes for devices without the adsorbed polymer (pristine) were produced by dipping electrodes with preadsorbed N719 into ethanol for 12 ± 2 h.

The photoanodes and counter electrodes were sandwiched with 60 μm Surlyn gaskets and sealed with a hot press. The cavity of the device was filled with the electrolyte, and the injection holes were sealed with a 25 μm Surlyn film and lamellar glass on top. Batches of DSSCs were prepared using two commercial electrolytes: one for high-performing devices based on the ACN solvent (EL-HPE) and one for highly stable devices with the 3-methoxypropionitrile solvent (EL-HSE); both commercial electrolytes were obtained from GreatCell Solar. For the composition of the electrolytes, interested readers can consult the reference.^42^

### 2.3 Characterization

The current vs applied potential (*I*–*V*) responses of the cells were recorded using a Zennium (Zahner) electrochemical station. The potential sweep rates were 50 and 5 mV s^–1^ for measurements under simulated solar and artificial light, respectively. Solar Simulator MiniSol (LSH-7320, Newport) (AM 1.5G filter, 100 mW cm^–2^) was used to provide standardized light flux. The power of the incident light flux was additionally controlled with the calibrated reference cell made of Si. The LED lamp (Osram, Class A+, 60 W, 2700 K) with emission spectra presented in Figure S4 was a typical indoor light source. The illuminance (lx) and light power (μW m^–2^) of the LED lamp were controlled with a calibrated Delta Ohm HD 2102.2 radiometer. The artificial light incident power was 149 and 275 μW·cm^–2^ at an illuminance of 500 and 1000 lx, respectively. For each DSSC type, a batch of 5 devices was produced, and the *I*–*V* response of each cell was obtained. Devices show a regular interval of photovoltaic metrics within ca. 5% of the average. In cases where a deviation of more than 5% was observed, the devices were excluded. *I*–*V* characterization under simulated solar light was performed with and without an aperture mask (circular-shaped 0.1 cm^2^); under artificial light, *I*–*V* curves were obtained without an aperture mask.

The electrochemical impedance spectra were collected by using an Autolab (PGSTAT 302 N, Metrohm) electrochemical station. Impedance spectra were recorded in the dark at a potential 20 mV below the open-circuit potential of the DSSCs; a sinusoidal perturbation with a peak-to-zero amplitude of 10 mV in the frequency range 100 kHz to 0.1 Hz was applied. The spectra were fitted using ZView software.

A Shimadzu UV-3600 spectrometer was used to obtain light absorption spectra of the transparent mesoporous TiO_2_ layer with the adsorbed N719 dye and polymers.

Infrared spectra were recorded at room temperature utilizing a VERTEX 70 Fourier transform infrared (FTIR) spectrometer (Bruker) in transmittance mode equipped with a DLaTGS detector. The measurements were conducted in attenuated total reflection (ATR) mode using a A225/Q Platinum ATR Diamond crystal with a single reflection accessory.

The setup for recording the incident photon conversion efficiency (IPCE) spectra included a 300 W Xe lamp, a Cornerstone 74125 monochromator, and a Merlin 70104 lock-in amplifier with a minimum sync frequency of 8 Hz. The system utilized optical filters and a light chopper. Calibration of the incident monochromatic light flux was performed with a Newport 70356 Si detector. For the bias light, a 100 W halogen lamp was used. The chopping frequency for the monochromatic light was set at 8.3 Hz. IPCE spectra were recorded without and with an aperture mask (0.1 cm^2^).

The lifetimes of the photoexcited states were monitored by time-correlated single-photon counting (TCSPC) using a FLS980 spectrometer (Edinburgh Instruments). Excitation was carried out with a pulsed laser diode at 475 nm, operating at 5 MHz, with ca. 80 ps pulse width. The instrument response was 90 ps fwhm as measured on quartz glass with BaSO_4_. The detector is based on a microchannel plate photomultiplier tube (MCP-PMT, cooled to −30 °C to eliminate noise) Hamamatsu detector set after the first emission monochromator. A 515 nm long pass filter was introduced prior to the emission monochromators to reject light scattering from the DSSC devices. The emission was monitored at 770 nm with a 10 nm bandwidth. The numerical analysis of the excited-state lifetime was determined after reconvolution of the photoluminescence decay, considering the instrumental response function. The measurements were performed on devices without scattering layers and under open-circuit conditions.

An Atlas SUNTEST XLS+ climatic chamber was used to assess the long-term photovoltaic stability of the devices according to the modified ISOS-L2 protocol^42^ under continuous soaking of simulated solar light; a Xe lamp with an AM 1.5G filter and a 380 nm UV cutoff filter was used as a light source. The intensity of the incident light was controlled using an internal Si detector and adjusted at 900 W m^–2^; the temperature in the camera was monitored using a blackbody detector and stabilized at 53 ± 3 °C.

## 3 Results and Discussion

### 3.1 Photovoltaic Performance of DSSCs with Polymer-Passivated Photoanodes

**Figure 2:**
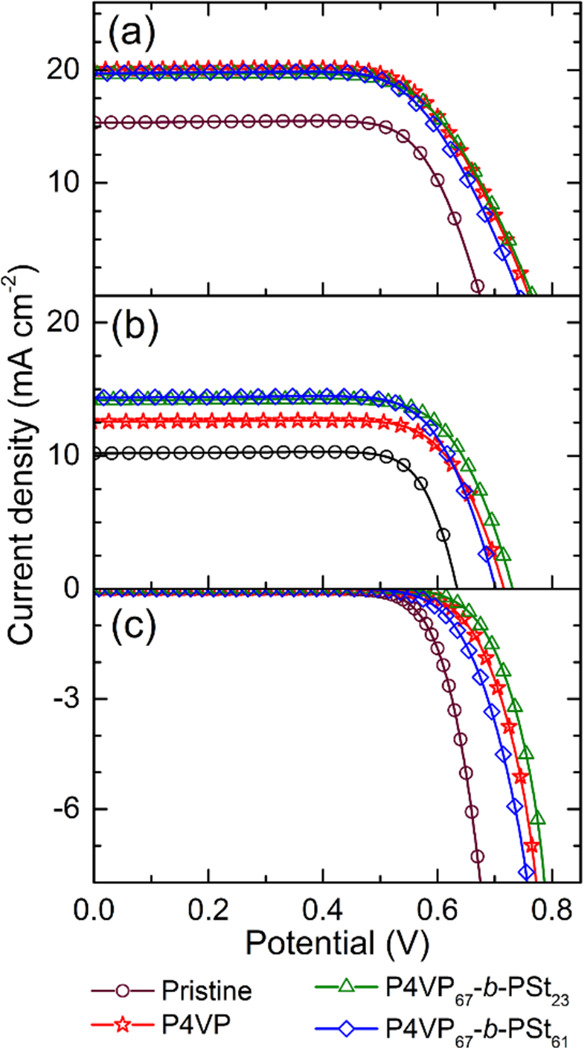
*I*–*V* curves of DSSCs produced with the pristine TiO_2_/N719 photoanode and with adsorbed P4VP or P4VP_67_-*b*-PSt_*x*_ polymers: photocurrent under AM 1.5G (100 mW cm^–2^) (a) without and (b) with the aperture mask. (c) Plot showing the current density in the dark.

Figure 2a,b shows *I*–*V* curves of the devices illuminated without and with an aperture mask, respectively. While determining the photoresponse of cells with an aperture mask is mandatory to obtain standardized metrics, the photoresponse from a fully illuminated device approximates its operation in real-world conditions, such as in a module arrangement, where the aperture mask is not applied.

Under both illumination conditions, the cells show an *I*–*V* response typical of a photodiode. The cells generate approximately 1.3–1.6 times more photocurrent when the entire device is exposed to incident light; the photoanode harvests the light scattered and reflected from the glass surfaces of the cell. The corresponding photovoltaic parameters were calculated from the *I*–*V* curves and are summarized in Table 1.

**Table 1:**
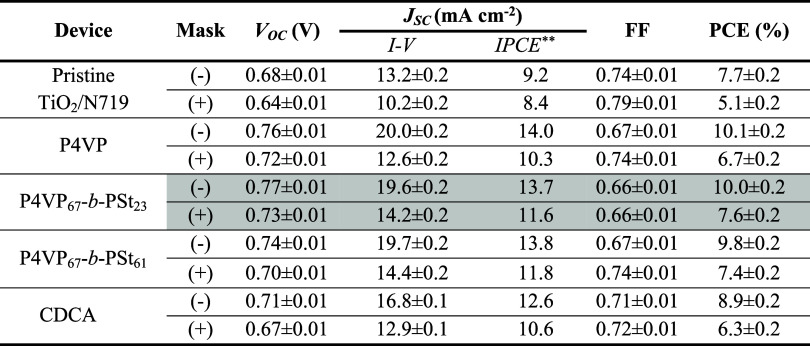
Photovoltaic Metrics* under AM 1.5G Illumination of DSSCs with Pristine TiO_2_/N719 Photoanodes and Treated with Coadsorbents^a^

* The photovoltaic metrics of the best cells with the photoanode passivated with the copolymer are marked in gray.

** Values of the *J*_SC_ obtained from the IPCE spectrum.

a The metrics were obtained from the *I*–*V* response and IPCE measurements without (−) and with (+) aperture masks.

Adsorption of P4VP and P4VP_67_-*b*-PSt_*x*_ polymers noticeably improves the open-circuit potential (*V*_OC_) and short-circuit current density (*J*_SC_) compared to their counterparts with untreated TiO_2_/N719 photoanodes. Polymeric coadsorbents cause a similar effect on the *I*–*V* response and IPCE as CDCA (Figure S5). Overall PCE values of 7.6 and 10.0% with and without the aperture mask, respectively, are obtained in the P4VP_67_-*b*-PSt_23_ devices. The other two coadsorbents also lead to comparable and reasonable PCE improvements (Table 1). Increasing ca. 3 times the *M*_W_ of the PSt fragment (P4VP_67_-*b*-PSt_23_ vs P4VP_67_-*b*-PSt_61_) leads to a modest reduction in the *J*_SC_ and *V*_OC_. This occurs because of insufficient passivation of the titania surface (as shown below with the EIS study). Due to a bulky PSt chain, adsorption from ethanol solution and packaging on TiO_2_ proceed less efficiently than a counterpart with a lower *M*_W_; the *M*_W_ of the adsorbed polymer and its concentration in the solution must be meticulously optimized to achieve the ideal balance between sufficient suppression of back electron recombination and avoiding overpassivation of the surface, as reported elsewhere.^38^

The PCEs achieved with polymeric coadsorbents are similar to those of devices with conventional coadsorbent CDCA. Cells with CDCA report an average PCE of 8.3% across ∼300 cases,^37^ with landmarks of 10.2^43^ and 11.3%^44^ in devices employing fine-tuned light-scattering and blocking layers, respectively.

**Figure 3:**
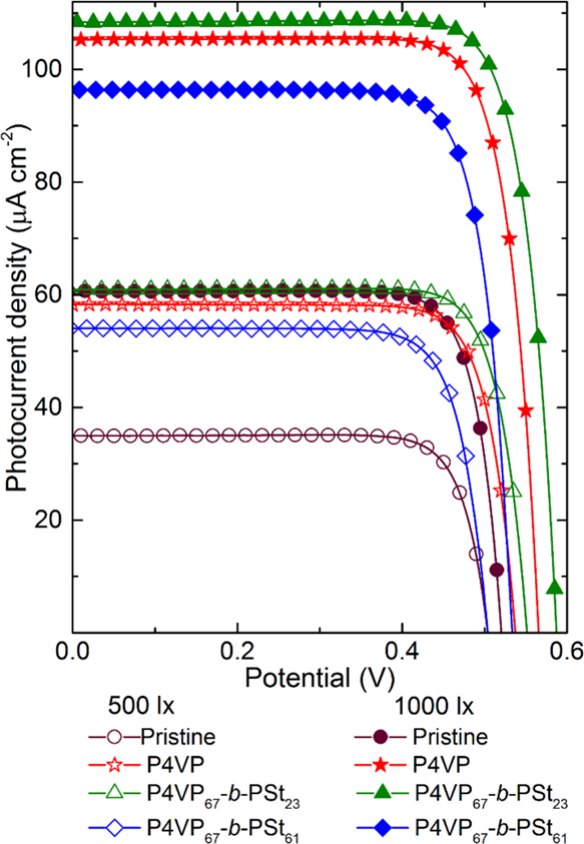
*I*–*V* curves of DSSCs with photoanodes passivated with P4VP_67_-*b*-PSt_*x*_ copolymers and the P4VP homopolymer obtained under different intensities of LED illumination.

Motivated by the emerging demand for indoor PV, the efficacy of the DSSCs with polymeric coadsorbents was tested for the conversion of artificial indoor light; Figure 3 shows the *I*–*V* response of the devices under the light of a typical LED indoor lamp. The PCE of the cells was determined as the ratio between the maximum output power of the device (*P*_out_) and the incident light power (*P*_in_). Table 2 shows the detailed metrics of the cells.

**Table 2:**
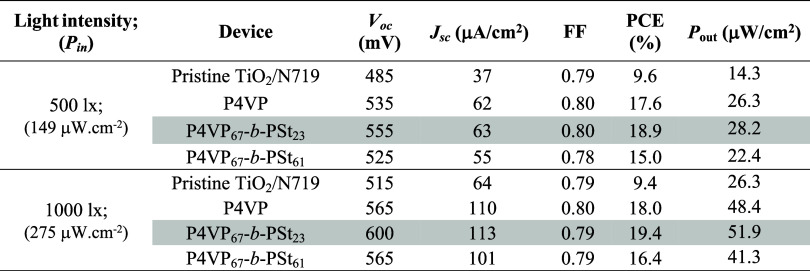
Photovoltaic Parameters* of DSSCs under LED Light

* The metrics of the devices with the most effective coadsorbent are marked in gray.

For all DSSCs tested, the saturation current density and *J*_SC_ increase 1.8-fold for the same increase in the incident light power. Such a behavior is commonly observed in DSSCs at low light intensities and can be explained by the fact that the low intensity of the incident light greatly reduces the number of electrons in the photoanode and the probability of their recombination. As a result, the *J*_SC_ is approximately proportional to the artificial light flux since the losses for the recombination of electrons are marginal.^19^ Passivation of photoanodes with the P4VP_67_-*b*-PSt_23_ copolymer leads to the best-performing cells; the highest *P*_out_ values were obtained at 28.2 and 51.9 μW cm^–2^, corresponding to the PCE values of 18.9 and 19.4% at 500 and 1000 lx, respectively.

**Figure 4:**
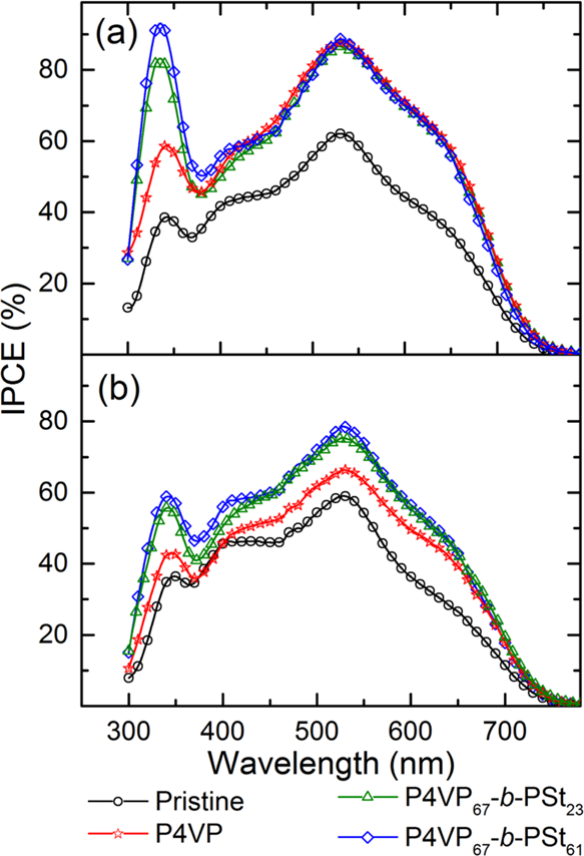
IPCE spectra of DSSCs recorded (a) without and (b) with the aperture mask.

The improvement in the overall PCE under 1 sun and dim artificial light after polymer adsorption is due to the higher *V*_OC_ and *J*_SC_ (Tables 1 and 2) and suppressed reverse current (Figure 2c). Figure 4 presents the IPCE spectra of the devices.

The IPCE onset below 760 nm is attributed to the transition from the ground state of the N719 dye to its lowest excited state, localized on TiO_2_ (1.58 eV).^45^ Upon coadsorption of polymers, the IPCE increases across all effective wavelength regions, with a significant improvement observed around 600–650 nm. This enhancement is likely due to dye disaggregation and an increased injection efficiency.

The *J*_SC_ value derived from the IPCE aligns with the values obtained from the *I*–*V* curves (Table 1). However, these values are approximately 28–30% lower, which can be due to reflection and absorption losses in FTO glass.^46^ Additionally, and more importantly, the IPCE is strongly affected by the delayed response of DSSCs to chopped monochromatic light (8.3 Hz in this study), as reported.^47^

**Figure 5:**
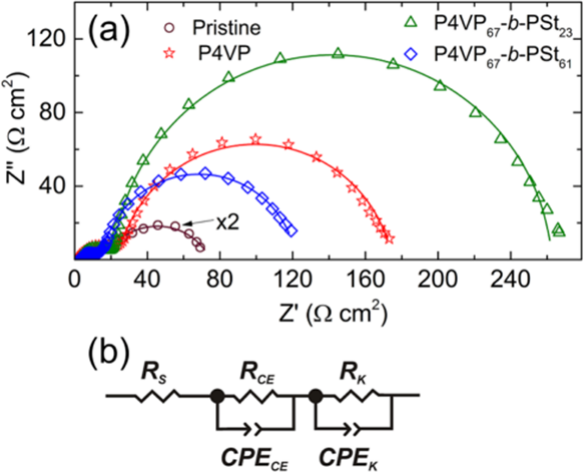
(a) Impedance response of DSSCs with the pristine TiO_2_/N719 photoanode and after coadsorption of polymers. (b) Equivalent electrical circuit used to fit the spectra. Solid lines show fittings to the equivalent circuit. Spectra were recorded in the dark at a potential 20 mV below the *V*_OC_.

An examination of the devices by EIS (Figure 5) shows that an increase in the charge transfer resistance at the photoanode/electrolyte interface after polymer adsorption is one of the key factors for the improvement in the photovoltaic performance and PCE.

Nyquist plots of the EIS response show two well-defined capacitive features. The small and large semicircles represent the electron transport at the interfaces between the counter electrode/electrolyte and the photoanode/electrolyte, respectively. The impedance spectra are consistent with the equivalent circuit, as shown in Figure 5b; the resistance *R*_S_ is the series resistance and *R*_CE_ and *R*_K_ are the resistances of charge transport at the interfaces of the counter electrode and photoanode with the electrolyte, respectively. CPE_CE_ and CPE_K_ are the respective constant phase elements. The resistances of the devices with and without coadsorbents are listed in Table 3.

**Table 3:** Resistances *R*_S_, *R*_CE_, and *R*_K_ Obtained by Fitting the Model to the Impedance Spectra

device	*R*_S_ (Ω·cm^2^)	*R*_CE_ (Ω·cm^2^)	*R*_K_ (Ω·cm^2^)
pristine TiO_2_/N719	2.4	7.0	28.0
P4VP	2.8	21.8	150.3
P4VP_67_-*b*-PSt_23_	2.7	19.1	240.9
P4VP_67_-*b*-PSt_61_	2.8	12.6	106.7

The series resistance *R*_S_ of the devices naturally remains constant; the dispersion of *R*_CE_ values is 4–8 Ω, which is negligible to affect the photovoltaic performance. *R*_K_ increases significantly, raising the charge transfer resistance at the photoanode/electrolyte interface from tens of ohm for pristine TiO_2_/N719 to several kΩ after polymer adsorption; back electron recombination is suppressed, leading to the observed increase in the *V*_OC_ and *J*_SC_. The *R*_K_ value is the lowest when using the copolymer with a higher number of repeating styrene units (P4VP_67_-*b*-PSt_61_) compared to other coadsorbents. This accounts for the lower *V*_OC_ observed in the device with the photoanode treated with P4VP_67_-*b*-PSt_61_, relative to those treated with P4VP_67_-*b*-PSt_23_ and P4VP coadsorbents. (Tables 1 and 2). A lower *R*_K_ also results in a higher dark current (Figure 2c). It is essential to recognize that poly(styrene) exhibits low solubility in ethanol and the solubility of P4VP_67_-*b*-PSt_*x*_ significantly decreases with the length of PSt. For instance, even 30 μM solutions of P4VP_67_-*b*-PSt_61_ in ethanol appear to be turbid, suggesting the beginning of polymer aggregation. In contrast, the P4VP_67_-*b*-PSt_23_ polymer does not display this issue. Likely due to its poor solubility in ethanol, a smaller amount of P4VP_67_-*b*-PSt_61_ is adsorbed onto the photoanode, leading to a lower *R*_K_ value.

**Figure 6:**
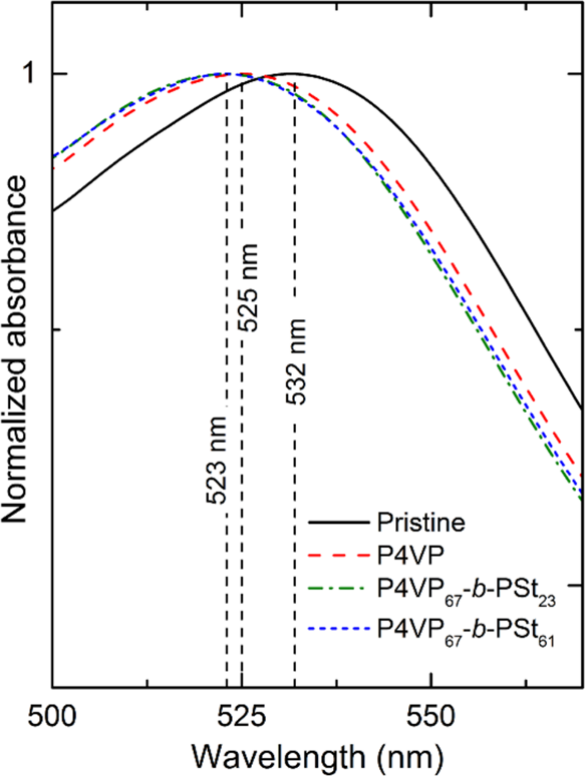
Normalized absorption spectra of the pristine TiO_2_/N719 layer and after coadsorption of the polymers.

In addition to the increase of the interfacial charge transfer resistance on the photoanode, upon adsorption of the polymers, a blue shift of the metal-to-ligand charge transfer optical band of the N719 dye is observed (Figure 6).

**Figure 7:**
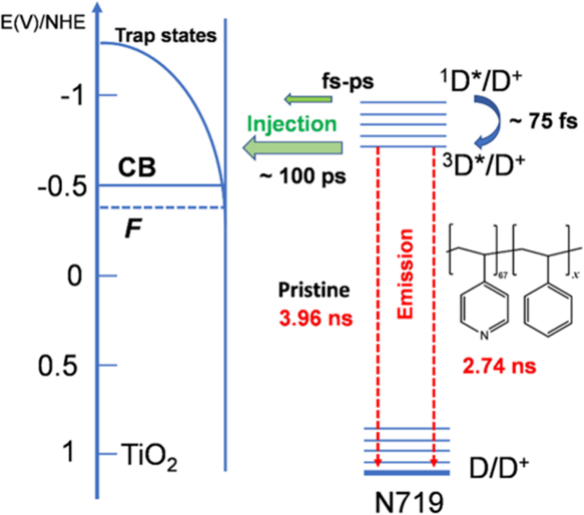
Simplified energy-kinetic diagram for electron injection from N719 to titania. The energy levels are presented with respect to the normal hydrogen electrode; plotted with the use of data of refs (48,49) and TCSPC data obtained in this study.

The absorption band at ca. 532 nm in TiO_2_/N719 is associated with π–π* and dπ–π charge transfer in N719 with the formation of singlet (^1^D*) and triplet (^3^D*) excited dye states, respectively.^48,49^ The transition from 1D* to 3D* excited states and the injection from 1D* to TiO_2_ are fast and occur within the fs range (Figure 7). However, the lifetime of the triplet state is much longer (∼10 ns) compared to 1D* (∼0.1 ps), making electron injection from 3D* to titania CB/trap states the major pathway for the photoelectron transfer to TiO_2_.^48^ Upon treatment with the polymers, the 532 nm band is blue-shifted to about 523–525 nm. The shift is ascribed to an increase in the energy of the lowest unoccupied molecular orbital (LUMO) of the ligand, causing π–π* transitions to occur at higher energies.^50^

**Figure 8:**
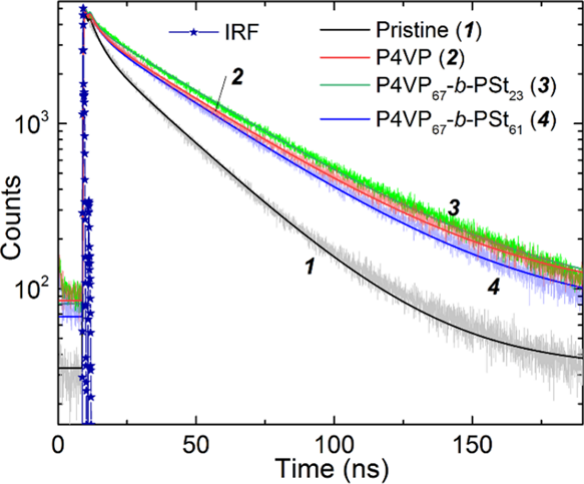
Time-resolved photoluminescence decay between devices with the pristine photoanode and with coadsorbed polymers. Bold lines stand for the fit results using a biexponential function. The instrument response function (IRF) is plotted with “star” symbols.

Time-correlated single-photon counting (TCSPC) was performed for evaluation of the N719 excited-state lifetime in the self-assembled monolayer (SAM) (Figure 8).

Two contributions of similar amplitudes are consistently observed regardless of the photoanode architecture. The fastest component is attributed to the excited-state quenching of the dye due to partial carrier injection into TiO_2_ nanocrystals, whereas the longer decay is ascribed to the radiative dye deactivation on the self-assembled monolayer. Interestingly, the introduction of the polymer alters the overall kinetics without changing the relative fraction of the two components. On one hand, the presence of polymers slightly accelerates the charge injection, whereas it prolongs the dye excited states from 30 to ca. 40 ns. The fitting with a two-exponential function is presented in Table 4.

**Table 4:** Results from the Fitting of PL Decay of the Devices Using a Biexponential Function^a^

	τ_1_ (ns)	*A*_1_	τ_2_ (ns)	*A*_2_
pristine	3.963 (±0.005)	0.0564 (±0.0006)	30.343 (±0.001)	0.0900 (±0.0003)
P4PV	3.730 (±0.006)	0.0502 (±0.0007)	40.055 (±0.001)	0.0939 (±0.0003)
P4VP_67_-*b*-PSt_23_	2.739 (±0.009)	0.0495 (±0.0008)	41.150 (±0.001)	0.103 (±0.0002)
P4VP_67_-*b*-PSt_61_	4.019 (±0.004)	0.0521 (±0.0006)	38.605 (±0.001)	0.0926 (±0.0002)

a The half-time and amplitude of the two contributions are tabulated.

These findings suggest that the polymer affects the dye packing in the SAM in good agreement with the data presented based on UV–visible absorption spectroscopy and IPCE, and the combined results with TCSPC support that the polymer hinders molecular dye aggregation in light of the faster charge injection and lower the radiative recombination. This may indicate a better electronic coupling of the dye with titania. These results are also well aligned with the enhanced device performance under low-light and AM 1.5G conditions.

### 3.2 Long-Term Stability of the Devices

The long lifetime and stability of the photovoltaic properties of a PV device are critical for practical use and prospects of commercialization. The stability of third-generation PV devices is commonly tested based on ISOS protocols and recommendations, which are described in detail.^42^ Polymer-passivated devices were evaluated under two test conditions: ISOS-D1 over 300 h and ISOS-L2 over 1000 h. The first method corresponds to natural aging in the dark, known as “shelf” aging, which mimics postproduction storage. In the second method, the cells are exposed to continuous illumination to simulate working conditions.

**Figure 9:**
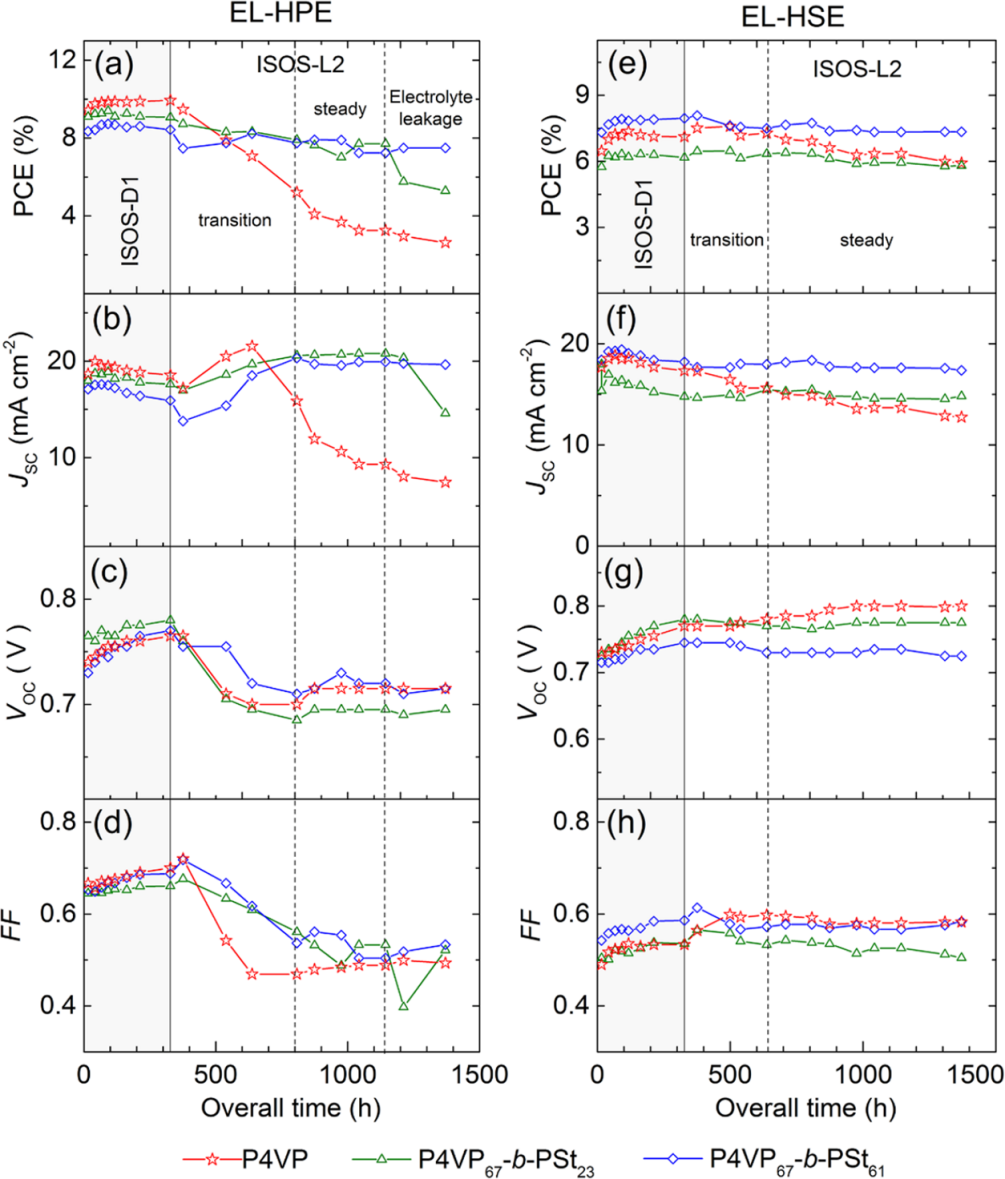
History of photovoltaic metrics (no aperture mask) of DSSCs with EL-HPE and EL-HSE electrolytes within ISOS-D1 (a gray area in the plot) and ISOS-L2 (a light area in the plot) testing protocols: (a, e) PCE; (b, f) *J*_SC_; (c, g) *V*_OC_; and (d, h) FF.

Figure 9 shows the evolution of the photovoltaic parameters of the DSSCs in both test protocols.

At the beginning of the shelf aging test, the PCE values of the unmasked devices passivated with P4VP, P4VP_67_-*b*-PSt_23_, and P4VP_67_-*b*-PSt_61_ using the EL-HPE electrolyte were 9.4 ± 0.2, 9.1 ± 0.2, and 8.4 ± 0.2%, respectively (the photovoltaic characteristics obtained with the aperture mask are presented in Table S2). The initial PCEs of the cells are within the regular scatter of the experimental values and are sufficient to evaluate the aging history of the devices with different recombination-suppressing additives. During the first ca. 200 h of storage in the dark, the PCE gradually increases (Figure 9a) regardless of the coadsorbent used for photoanode passivation. The improvement of the overall PCE is accompanied by an increase of the *V*_OC_ and FF (Figure 9c,d), while the *J*_SC_ slightly decreases. This phenomenon is quite complex, although commonly observed in DSSCs, and can be attributed to the balance of sorption/desorption processes of electrolyte components, dyes, and additives at the titania/dye/electrolyte interface.^51^ After about 300 h of aging in the dark, all photovoltaic metrics stabilize. Three clearly defined features can be seen in the progression chart. During the first ca. 470 h of light aging, all photovoltaic metrics change gradually (transition region); during the next 350 h, the DSSCs produce relatively constant metrics. After 1150 h of overall aging, the P4VP_67_-*b*-PSt_23_ devices show a decrease in the photovoltaic performance due to electrolyte leakage, making further evaluation of the device stability meaningless.

**Figure 10:**
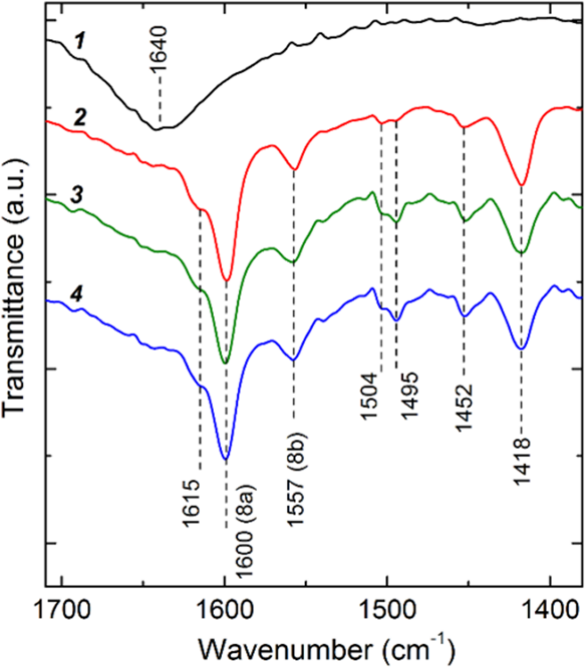
FTIR spectra of (1) pristine anatase nanoparticles and after immersing in an ethanol solution of (2) P4VP, (3) P4VP67-*b*-PSt23, and (4) P4VP67-*b*-PSt61.

After 470 h of light aging, the *V*_OC_ decreases to ca. 50 mV and the FF drops from 0.7 to about. 0.5, but the *J*_SC_ displays an increase. Such an initial behavior in photovoltaic metrics with light aging is commonly observed in I_3_^–^/3I^–^ devices when electrolytes (EL-HPE) with a low concentration of I_3_^–^ ions are used. In a continuously operating cell, the electrolyte becomes depleted of I_3_^–^ ions until its concentration stabilizes.^52^ A decrease in the I_3_^–^ ion concentration leads to an upward shift in the redox potential of the electrolyte and a decrease in the *V*_OC_. At the same time, the probability of electron recombination decreases, leading to a higher *J*_SC_. The FF drop is primarily due to the increase in the Nernst diffusion impedance at lower triiodide concentrations.^53^ After about 300 h of the light soaking test, the *V*_OC_, FF, and *J*_SC_ of the devices passivated with P4VP_67_-*b*-PSt_*x*_ polymers stabilize, leading to a constant PCE. The overall PCE drop after 1140 h of aging is 15.4 and 13% compared to the initial values in the cells with P4VP_67_-*b*-PSt_23_ and P4VP_67_-*b*-PSt_61_ coadsorbents, respectively. P4VP-passivated devices show a PCE drop of 65.6%, which can be attributed to a strong *J*_SC_ drop. The low stability of P4VP-passivated photoanodes is mainly due to two factors. The first reason is the weak chemical bonding of the pyridine moieties to titanium dioxide via the coordination of the nitrogen atom with the Lewis acid sites of the titania surface.^38,54−56^ All three studied polymers exhibit characteristic signatures of pyridine coordination with titania Lewis centers upon adsorption on TiO_2_ (Figure 10).

For unmodified titania, a broad absorption band around 1640 cm^–1^ is indicative of OH vibrations stemming from water both chemically and physically bound to the surface of TiO_2_.^57,58^ After submerging TiO_2_ in polymer solutions, FTIR spectroscopy reveals several distinct bands between 1400–1700 cm^–1^. The band at 1452 cm^–1^ is due to the planar deformation vibrations of the CH_2_ groups in the polymer chains.^59^ The bands at 1599, 1557, 1495, and 1418 cm^–1^ correspond to the in-plane vibrational modes 8a, 8b, 19a, and 19b of the pyridine ring, respectively.^54,55^ The band at 1615 cm^–1^ points to the formation of coordinative bonds between the nitrogen atoms in the pyridine units and the Lewis acid centers of TiO_2_.^60^ Additionally, the absorption bands at 1452 and 1495 cm^–1^ are associated with C=C stretching in the benzene ring, which is notably pronounced in adsorbed block copolymers.

The calculated heat of adsorption of pyridine on TiO_2_ is only ca. 70 kJ/mol^61^ (the heat of absorption of the N719 dye on TiO_2_ is ca. 190 kJ/mol,^62^ yet heat- and light-induced desorptions of the N719 dye are commonly known problems affecting the stability of DSSCs).^63^

The second reason is the good solubility of P4VP in ACN-based electrolytes. The low chemical binding to titanium dioxide and high solubility in the electrolyte lead to the desorption of P4VP;^64^ back electron recombination is facilitated, leading to the observed decrease in the current, *V*_OC_, and the overall PCE. The superior stability of the P4VP_67_-*b*-PSt_*x*_ coadsorbents on the titania surface is apparently due to the added PSt fragments. PSt is insoluble in ACN; the incompatibility of the polymer’s PSt fragments with the ACN electrolyte causes the polymer to remain on titanium dioxide, preventing desorption and leading to the stable suppression of back electron recombination. Although the long-term photovoltaic performance of the devices with P4VP_67_-*b*-PSt_23_ and P4VP_67_-*b*-PS_61_ coadsorbents is quite similar, the polymer with the longer PSt chain enables more stable PV metrics.

To our surprise, a good stability of the devices with P4VP_67_-*b*-PSt_*x*_ copolymers in ACN-based electrolytes was observed. At the end of the aging tests, the overall PCE of the cells with P4VP_67_-*b*-PSt_23_ and P4VP_67_-*b*-PSt_61_ coadsorbents was 6.4 and 7.1%, respectively, when calculated without the aperture mask and 5.8 and 5.9%, respectively, with the aperture mask (Table S2). It should be noted that a longer stability evaluation was limited by extrinsic degradation of the device, i.e., electrolyte leakage. This should be addressed in the next studies by implementing a more robust encapsulation.^64^ Full encapsulation with glass, which provides the highest possible degree of hermeticity, is already an available option for DSSCs;^65,66^ extrinsic degradation factors caused by electrolyte leakage, oxygen ingress, or moisture are eliminated, allowing the fabrication of stable liquid junction cells. Another option to extend the lifetime of the cells and study their long-term stability is to use electrolytes with a lower volatility, which we further investigated (Figure 9e,f).

The long-term stability of DSSCs with 3-methoxypropionitrile-based electrolytes (EL-HSE) was evaluated under the same aging conditions. The EL-HSE electrolyte leads to a slightly lower PCE of the cells, which is a typical drawback in the search for an appropriate balance between high stability and the initial PCE.^41^ DSSCs with all three polymers show acceptable initial PCE values (Table S3), but with an expectedly lower initial PCE of 5.8% with the P4VP_67_-*b*-PSt_23_ polymer, mainly due to the lower current. During aging in the dark, the evolution of the photovoltaic metrics generally follows those of the counterparts with EL-HPE electrolytes. The time at which the device transitions to a stable photovoltaic response under light exposure is ca. 300 h for the cells passivated with block copolymers, with the P4VP devices showing a continuous and significant decrease in the *J*_SC_ and PCE throughout the test period. DSSCs with P4VP_67_-*b*-PSt_*x*_ coadsorbents demonstrated a stable PCE progression and maintained their initial PCE after 1000 h of continuous operation under simulated sunlight; this makes them very attractive and effective recombination suppressors aimed at fabricating durable and efficient devices.

The observed good stability of polymer-passivated N719/triiodide DSSCs aligns well with previous studies,^27,41,67^ which also suggest, among other factors, the importance of properly selected coadsorbents, electrolyte additives, and mitigating extrinsic degradation factors, such as electrolyte escape or water ingress through the sealing,^66^ to achieve a representative lifetime of the devices.

P4VP and PS are robust polymers that notably degrade or undergo molecular structure changes under pyrolysis temperatures or prolonged UV radiation, neither of which applies to ISOS-L2 testing conditions. During the long-term operation with volatile ACN, performance metrics are degraded primarily due to electrolyte evaporation. However, using a higher-boiling-point solvent mitigates this issue, resulting in a stable device over a testing period exceeding 1000 h. Given the significant impact of coadsorbed polymers on device metrics compared to pristine counterparts, it is logical to conclude that the polymer structure is mostly preserved under light exposure during the testing period. Nevertheless, partial photooxidation of the polymers or formation of molecular iodine/polymer complexes^36,68^ cannot be excluded in the triiodide environment, warranting a more detailed stability assessment and deep examination of molecular dynamics during long-term operation for future research.

Further studies could focus on evaluating the effectiveness and stability of the polymeric recombination suppressors in cells sensitized with emerging dyes,^69,70^ dyes attractive for indoor light conversion,^70^ advanced electrolytes based on Co(III/II) and Cu(II/I) complexes, and in solid-state DSSCs.

## 4 Conclusions

Block copolymers PSt-P4VP_67_-*b*-PSt_(*x*=23;61)_ were prepared by RAFT polymerization and disclosed as efficient promoters of electron injection from the excited dye to titania, increasing the excited states’ lifetime and suppressing back electron recombination. Adsorption of the polymers on the TiO_2_/N719 photoanode drastically increases the charge transport resistance at the interface between the photoanode and the electrolyte and promotes stronger anchoring bonds of the dye to titania. Under 1 sun, the DSSCs with P4VP_67_-*b*-PSt_23_ and P4VP_67_-*b*-PSt_61_-treated photoanodes showed PCEs of 9.8–10.1%, outperforming the counterparts with the untreated photoanode and 7.7% of the PCE. Under 1000 lx light, P4VP_67_-*b*-PSt_23,_ and P4VP_67_-*b*-PSt_61_ cells achieved PCE values of 19.4 and 16.4%, respectively, with an output power of 51.9 and 41.3 μW·cm^–2^, respectively; this is a promising performance for use as indoor PVs.

P4VP_67_-*b*-PSt_*x* (*x*=23;61)_ enables highly stable DSSCs with ACN or 3-methoxypropionitrile electrolytes, as determined under standard ISOS-D1 and ISOS-L2 test conditions. The permanent recombination suppression with P4VP_67_-*b*-PSt_*x* (*x*=23;61)_ coadsorbents is due to the insolubility of PSt in the DSSC electrolytes; the incompatibility of the PSt fragments with the DSSC electrolyte prevents the desorption of the polymer. The P4VP_67_-*b*-PSt_*x* (*x*=23;61)_ coadsorbents enable the fabrication of DSSC devices that maintain their high initial PCE after 300 h of aging in the dark and 1000 h of continuous operation under simulated sunlight.

## Supporting Information Available

Synthesis of block copolymers; ^1^H NMR spectra of P4VP and P4VP-*b*-PSt copolymers; macromolecular characteristics of P4VP MacroCTA and P4VP-*b*-PSt; SEC traces of polymers; scanning electron microscopy (SEM) images of the photoanode cross-section; the emission spectrum of an LED lamp; photocurrent and IPCE of DSSCs with CDCA; and photovoltaic metrics of DSSCs (PDF)

## References

[ref1] Muñoz-García A. B.; Benesperi I.; Boschloo G.; Concepcion J. J.; Delcamp J. H.; Gibson E. A.; Meyer G. J.; Pavone M.; Pettersson H.; Hagfeldt A.; Freitag M. Dye-sensitized solar cells strike back. Chem. Soc. Rev. 2021, 50 (22), 12450–12550. 10.1039/D0CS01336F. 34590638 PMC8591630

[ref2] Kokkonen M.; Talebi P.; Zhou J.; Asgari S.; Soomro S. A.; Elsehrawy F.; Halme J.; Ahmad S.; Hagfeldt A.; Hashmi S. G. Advanced research trends in dye-sensitized solar cells. J. Mater. Chem. A 2021, 9 (17), 10527–10545. 10.1039/D1TA00690H. PMC8095349 33996094

[ref3] Barichello J.; Mariani P.; Vesce L.; Spadaro D.; Citro I.; Matteocci F.; Bartolotta A.; Di Carlo A.; Calogero G. Bifacial dye-sensitized solar cells for indoor and outdoor renewable energy-based application. J. Mater. Chem. C 2024, 12 (7), 2317–2349. 10.1039/D3TC03220E.

[ref4] Zhang D.; Stojanovic M.; Ren Y.; Cao Y.; Eickemeyer F. T.; Socie E.; Vlachopoulos N.; Moser J.-E.; Zakeeruddin S. M.; Hagfeldt A.; Grätzel M. A molecular photosensitizer achieves a V_oc_ of 1.24 V enabling highly efficient and stable dye-sensitized solar cells with copper(II/I)-based electrolyte. Nat. Commun. 2021, 12, 1777 10.1038/s41467-021-21945-3. 33741953 PMC7979847

[ref5] Ren Y.; Zhang D.; Suo J.; Cao Y.; Eickemeyer F. T.; Vlachopoulos N.; Zakeeruddin S. M.; Hagfeldt A.; Grätzel M. Hydroxamic Acid Preadsorption Raises Efficiency of Cosensitized Solar Cells. Nature 2023, 613, 60–65. 10.1038/s41586-022-05460-z. 36288749

[ref6] Masud N.; Zhou H.; Kim H. K. Minimization of Photovoltage Loss of Iodine Electrolytes by Ethylene Carbonate and PAN-Based Block Copolymer for High-Performance Quasi-Solid-State Organic Dye-Sensitized Solar Cells. ACS Appl. Polym. Mater. 2023, 5 (11), 9671–9680. 10.1021/acsapm.3c02272.

[ref7] Chalkias D. A.; Charalampopoulos C.; Andreopoulou A. K.; Karavioti A.; Stathatos E. Spectral Engineering of Semi-Transparent Dye-Sensitized Solar Cells Using New Triphenylamine-Based Dyes and an Iodine-Free Electrolyte for Greenhouse-Oriented Applications. J. Power Sources 2021, 496, 229842 10.1016/j.jpowsour.2021.229842.

[ref8] Ursu D.; Vajda M.; Miclau M. Highly Efficient Dye-Sensitized Solar Cells for Wavelength-Selective Greenhouse: A Promising Agrivoltaic System. Int. J. Energy Res. 2022, 46 (13), 18550–18561. 10.1002/er.8469.

[ref9] Fagiolari L.; Sampò M.; Lamberti A.; Amici J.; Francia C.; Bodoardo S.; Bella F. Integrated Energy Conversion and Storage Devices: Interfacing Solar Cells, Batteries and Supercapacitors. Energy Storage Mater. 2022, 51 (6), 400–434. 10.1016/j.ensm.2022.06.051.

[ref10] Khataee A.; Azevedo J.; Dias P.; Ivanou D.; Dražević E.; Bentien A.; Mendes A. Integrated Design of Hematite and Dye-Sensitized Solar Cell for Unbiased Solar Charging of an Organic-Inorganic Redox Flow Battery. Nano Energy 2019, 62, 832–843. 10.1016/j.nanoen.2019.06.001.

[ref11] Kang S. H.; Jeong M. J.; Eom Y. K.; Choi I. T.; Kwon S. M.; Yoo Y.; Kim J.; Kwon J.; Park J. H.; Kim H. K. Porphyrin Sensitizers with Donor Structural Engineering for Superior Performance Dye-Sensitized Solar Cells and Tandem Solar Cells for Water Splitting Applications. Adv. Energy Mater. 2017, 7, 1602117 10.1002/aenm.201602117.

[ref12] da Silva Lopes T.; Dias P.; Monteiro R.; Vilanova A.; Ivanou D.; Mendes A. A 25 cm^2^ Solar Redox Flow Cell: Facing the Engineering Challenges of Upscaling. Adv. Energy Mater. 2022, 12, 2102893 10.1002/aenm.202102893.

[ref13] Tavares A. P. M.; Truta L. A. A. N. A.; Moreira F. T. C.; Carneiro L. P. T.; Sales M. G. F. Self-Powered and Self-Signalled Autonomous Electrochemical Biosensor Applied to Cancinoembryonic Antigen Determination. Biosens. Bioelectron. 2019, 140, 111320 10.1016/j.bios.2019.111320. 31150987

[ref14] Bandara T. M. W. J.; Hansadi J. M. C.; Bella F. A Review of Textile Dye-Sensitized Solar Cells for Wearable Electronics. Ionics 2022, 28, 2563–2583. 10.1007/s11581-022-04582-8.

[ref15] Ding Y.; Wang Z.; Duan X.; Liu R. Flexible photo-charging power sources for wearable electronics. Mater. Today Energy 2023, 33, 101276 10.1016/j.mtener.2023.101276.

[ref16] Aslam A.; Mehmood U.; Arshad M. H.; Ishfaq A.; Zaheer J.; UI Haq Khan A.; Sufyan M. Dye-sensitized solar cells (DSSCs) as a potential photovoltaic technology for the self-powered internet of things (IoTs) applications. Sol. Energy 2020, 207, 874–892. 10.1016/j.solener.2020.07.029.

[ref17] Srivishnu K. S.; Rajesh M. N.; Prasanthkumar S.; Giribabu L. Photovoltaics for indoor applications: Progress, challenges and perspectives. Sol. Energy 2023, 264, 112057 10.1016/j.solener.2023.112057.

[ref18] Masud; Zhou H.; Kim H. K. Effective redox shuttles for polymer gel electrolytes-based quasi-solid-state dye-sensitized solar cells in outdoor and indoor applications: Comprehensive comparison and guidelines. Mater. Today Energy 2023, 34, 101299 10.1016/j.mtener.2023.101299.

[ref19] Aftabuzzaman M.; Sarker S.; Lu C.; Kim H. K. In-depth understanding of the energy loss and efficiency limit of dye-sensitized solar cells under outdoor and indoor conditions. J. Mater. Chem. A 2021, 9, 24830–24848. 10.1039/D1TA03309C.

[ref20] Michaels H.; Rinderle M.; Freitag R.; Benesperi I.; Edvinsson T.; Socher R.; Gagliardi A.; Freitag M. Dye-sensitized solar cells under ambient light powering machine learning: towards autonomous smart sensors for the internet of things. Chem. Sci. 2020, 11 (11), 2895–2906. 10.1039/C9SC06145B. 34122790 PMC8157489

[ref21] Hagfeldt A.; Boschloo G.; Sun L.; Kloo L.; Pettersson H. Dye-Sensitized Solar Cells. Chem. Rev. 2010, 110 (11), 6595–6663. 10.1021/cr900356p. 20831177

[ref22] Manthou V. S.; Pefkianakis E. K.; Falaras P.; Vougioukalakis G. C. Co-Adsorbents: A Key Component in Efficient and Robust Dye-Sensitized Solar Cells. ChemSusChem 2015, 8 (4), 588–599. 10.1002/cssc.201403211. 25650987

[ref23] Song B. J.; Song H. M.; Choi I. T.; Kim S. K.; Seo K. D.; Kang M. S.; Lee M. J.; Cho D. W.; Ju M. J.; Kim H. K. A desirable hole-conducting coadsorbent for highly efficient dye-sensitized solar cells through an organic redox cascade strategy. Chem. - Eur. J. 2011, 17 (40), 11115–11121. 10.1002/chem.201100813. 21922547

[ref24] Choi I. T.; Ju M. J.; Song S. H.; Kim S. G.; Cho D. W.; Im C.; Kim H. K. Tailor-made hole-conducting coadsorbents for highly efficient organic dye-sensitized solar cells. Chem. - Eur. J. 2013, 19 (46), 15545–15555. 10.1002/chem.201301658. 24115151

[ref25] Song H. M.; Seo K. D.; Kang M. S.; Choi I. T.; Kim S. K.; Eom Y. K.; Ryu J. H.; Ju M. J.; Kim H. K. A simple triaryl amine-based dual functioned co-adsorbent for highly efficient dye-sensitized solar cells. J. Mater. Chem. 2012, 22 (9), 3786–3794. 10.1039/c2jm16021h.

[ref26] Kay A.; Grätzel M. Artificial Photosynthesis. 1. Photosensitization of TiO_2_ Solar Cells with Chlorophyll Derivatives and Related Natural Porphyrins. J. Phys. Chem. A 1993, 97 (23), 6272–6277. 10.1021/j100125a029.

[ref27] Lee K. M.; Chen C. Y.; Wu S. J.; Chen S. C.; Wu C. G. Surface Passivation: The Effects of CDCA Co-Adsorbent and Dye Bath Solvent on the Durability of Dye-Sensitized Solar Cells. Sol. Energy Mater. Sol. Cells 2013, 108, 70–77. 10.1016/j.solmat.2012.08.008.

[ref28] Salvatori P.; Marotta G.; Cinti A.; Anselmi C.; Mosconi E.; De Angelis F. Supramolecular Interactions of Chenodeoxycholic Acid Increase the Efficiency of Dye-Sensitized Solar Cells Based on a Cobalt Electrolyte. J. Phys. Chem. C 2013, 117 (8), 3874–3887. 10.1021/jp4003577.

[ref29] Friedrich D.; Valldecabres L.; Kunst M.; Moehl T.; Zakeeruddin S. M.; Grätzel M. Dye Regeneration Dynamics by Electron Donors on Mesoscopic TiO_2_ Films. J. Phys. Chem. C 2014, 118 (7), 3420–3425. 10.1021/jp4113206.

[ref30] Trilaksana H.; Shearer C.; Kloo L.; Andersson G. G. Restructuring of Dye Layers in Dye Sensitized Solar Cells: Cooperative Adsorption of N719 and Chenodeoxycholic Acid on Titania. ACS Appl. Energy Mater. 2019, 2 (1), 124–130. 10.1021/acsaem.8b01864.

[ref31] Hofmann A. F.; Hagey L. R. Key Discoveries in Bile Acid Chemistry and Biology and Their Clinical Applications: History of the Last Eight Decades. J. Lipid Res. 2014, 55 (8), 1553–1595. 10.1194/jlr.R049437. 24838141 PMC4109754

[ref32] Chandiran A. K.; Zakeeruddin S. M.; Humphry-Baker R.; Nazeeruddin M. K.; Grätzel M.; Sauvage F. Investigation on the Interface Modification of TiO_2_ Surfaces by Functional Co-Adsorbents for High-Efficiency Dye-Sensitized Solar Cells. ChemPhysChem 2017, 18 (19), 2724–2731. 10.1002/cphc.201700486. 28881086

[ref33] da Silva L.; Freeman H. Variation in Hydrophobic Chain Length of Co-Adsorbents to Improve Dye-Sensitized Solar Cell Performance. Phys. Chem. Chem. Phys. 2019, 21 (30), 16771–16778. 10.1039/C9CP02439E. 31328218

[ref34] Tseng Y. H.; Li C. T.; Huang G. W.; Chen Y. C.; Jeng R. J.; Dai S. A. Dendritic-Based Co-Adsorbents for Dye-Sensitized Solar Cells: Effect of the Generations and Alkyl Chain Lengths. Synth. Met. 2021, 274, 116711 10.1016/j.synthmet.2021.116711.

[ref35] Lee Y. G.; Park S.; Cho W.; Son T.; Sudhagar P.; Jung J. H.; Wooh S.; Char K.; Kang Y. S. Effective Passivation of Nanostructured TiO_2_ Interfaces with PEG-Based Oligomeric Coadsorbents to Improve the Performance of Dye-Sensitized Solar Cells. J. Phys. Chem. C 2012, 116 (11), 6770–6777. 10.1021/jp210360n.

[ref36] Lee Y. G.; Song D.; Jung J. H.; Wooh S.; Park S.; Cho W.; Wei W.; Char K.; Kang Y. S. TiO_2_ Surface Engineering with Multifunctional Oligomeric Polystyrene Coadsorbent for Dye-Sensitized Solar Cells. RSC Adv. 2015, 5 (84), 68413–68419. 10.1039/C5RA12889G.

[ref37] Hora C.; Santos F.; Sales M. G. F.; Ivanou D.; Mendes A. Conventional and Back-Illuminated Cobalt- and Iodine-Mediated Dye-Sensitized Solar Cells for Artificial and Solar Light Conversion. ACS Appl. Energy Mater. 2022, 5 (12), 14846–14857. 10.1021/acsaem.2c02307.

[ref38] Rodrigues D. F. S. L.; Santos F.; Abreu C. M. R.; Coelho J. F. J.; Serra A. C.; Ivanou D.; Mendes A. Passivation of the TiO_2_ Surface and Promotion of N719 Dye Anchoring with Poly(4-vinylpyridine) for Efficient and Stable Dye-Sensitized Solar Cells. ACS Sustainable Chem. Eng. 2021, 9 (17), 5981–5990. 10.1021/acssuschemeng.1c00842.

[ref39] Perrier S. 50th Anniversary Perspective: RAFT Polymerization - A User Guide. Macromolecules 2017, 50 (19), 7433–7447. 10.1021/acs.macromol.7b00767.

[ref40] Abreu C. M. R.; Fonseca A. C.; Rocha N. M. P.; Guthrie J. T.; Serra A. C.; Coelho J. F. J. Poly(vinyl chloride) by reversible deactivation radical polymerization: current status and future perspectives. Prog. Polym. Sci. 2018, 87, 34–69. 10.1016/j.progpolymsci.2018.06.007.

[ref41] Sauvage F. A Review on Current Status of Stability and Knowledge on Liquid Electrolyte-Based Dye-Sensitized Solar Cells. Adv. Chem. 2014, 2014, 939525 10.1155/2014/939525.

[ref42] Reese M. O.; Gevorgyan S. A.; Jørgensen M.; Bundgaard E.; Kurtz S. R.; Ginley D. S.; Olson D. C.; Lloyd M. T.; Morvillo P.; Katz E. A.; Elschner A.; Haillant O.; Currier T. R.; Shrotriya V.; Hermenau M.; Riede M.; Kirov K. R.; Trimmel G.; Rath T.; Inganäs O.; Zhang F.; Andersson M.; Tvingstedt K.; Lira-Cantu M.; Laird D.; McGuiness C.; Gowrisanker S.; Pannone M.; Xiao M.; Hauch J.; Steim R.; Delongchamp D. M.; Rösch R.; Hoppe H.; Espinosa N.; Urbina A.; Yaman-Uzunoglu G.; Bonekamp J. B.; Van Breemen A. J. J. M.; Girotto C.; Voroshazi E.; Krebs F. C. Consensus Stability Testing Protocols for Organic Photovoltaic Materials and Devices. Sol. Energy Mater. Sol. Cells 2011, 95 (5), 1253–1267. 10.1016/j.solmat.2011.01.036.

[ref43] Wang Z. S.; Kawauchi H.; Kashima T.; Arakawa H. Significant Influence of TiO_2_ Photoelectrode Morphology on the Energy Conversion Efficiency of N719 Dye-Sensitized Solar Cell. Coord. Chem. Rev. 2004, 248 (13–14), 1381–1389. 10.1016/j.ccr.2004.03.006.

[ref44] Thogiti S.; Park J. Y.; Thuy C. T. T.; Lee D. K.; Min B. K.; Yun H. J.; Kim J. H. High-Performance Dye-Sensitized Solar Cells through Graded Electron Transport in Band-Engineered W-TiO_2_ Cascade Layer. ACS Sustainable Chem. Eng. 2018, 6 (10), 13025–13034. 10.1021/acssuschemeng.8b02543.

[ref45] De Angelis F.; Fantacci S.; Mosconi E.; Nazeeruddin M. K.; Grätzel M. Absorption Spectra and Excited State Energy Levels of the N719 Dye on TiO_2_ in Dye-Sensitized Solar Cell Models. J. Phys. Chem. C 2011, 115 (17), 8825–8831. 10.1021/jp111949a.

[ref46] Ooyama Y.; Harima Y. Photophysical and Electrochemical Properties, and Molecular Structures of Organic Dyes for Dye-Sensitized Solar Cells. ChemPhysChem 2012, 13, 4032–4080. 10.1002/cphc.201200218. 22807392

[ref47] Guo X.-Z.; Luo Y.-H.; Zhang Y.-D.; Huang X.-C.; Li D.-M.; Meng Q.-B. Study on the Effect of Measuring Methods on Incident Photon-to-Electron Conversion Efficiency of Dye-Sensitized Solar Cells by Home-Made Setup. Rev. Sci. Instrum. 2010, 81, 103106 10.1063/1.3488456. 21034074

[ref48] Listorti A.; O’Regan B.; Durrant J. R. Electron Transfer Dynamics in Dye-Sensitized Solar Cells. Chem. Mater. 2011, 23 (15), 3381–3399. 10.1021/cm200651e.

[ref49] Klein M.; Pankiewicz R.; Zalas M.; Stampor W. Magnetic Field Effects in Dye-Sensitized Solar Cells Controlled by Different Cell Architecture. Sci. Rep. 2016, 6, 30077 10.1038/srep30077. 27440452 PMC4954973

[ref50] Singh J.; Gusain A.; Saxena V.; Chauhan A. K.; Veerender P.; Koiry S. P.; Jha P.; Jain A.; Aswal D. K.; Gupta S. K. XPS, UV-Vis, FTIR, and EXAFS Studies to Investigate the Binding Mechanism of N719 Dye onto Oxalic Acid Treated TiO_2_ and Its Implication on Photovoltaic Properties. J. Phys. Chem. C 2013, 117 (41), 21096–21104. 10.1021/jp4062994.

[ref51] Gao J.; El-Zohry A. M.; Trilaksana H.; Gabrielsson E.; Leandri V.; Ellis H.; D’Amario L.; Safdari M.; Gardner J. M.; Andersson G.; Kloo L. Light-Induced Interfacial Dynamics Dramatically Improve the Photocurrent in Dye-Sensitized Solar Cells: An Electrolyte Effect. ACS Appl. Mater. Interfaces 2018, 10 (31), 26241–26247. 10.1021/acsami.8b06897. 29996051

[ref52] Berginc M.; Krašovec U. O.; Topič M. Outdoor Ageing of the Dye-Sensitized Solar Cell under Different Operation Regimes. Sol. Energy Mater. Sol. Cells 2014, 120, 491–499. 10.1016/j.solmat.2013.09.029.

[ref53] Kato N.; Takeda Y.; Higuchi K.; Takeichi A.; Sudo E.; Tanaka H.; Motohiro T.; Sano T.; Toyoda T. Degradation Analysis of Dye-Sensitized Solar Cell Module after Long-Term Stability Test under Outdoor Working Condition. Sol. Energy Mater. Sol. Cells 2009, 93 (6–7), 893–897. 10.1016/j.solmat.2008.10.022.

[ref54] Ferwerda R.; Van Der Maas J. H.; Van Duijneveldt F. B. Pyridine Adsorption onto Metal Oxides: An Ab Initio Study of Model Systems. J. Mol. Catal. A: Chem. 1996, 104 (3), 319–328. 10.1016/1381-1169(95)00179-4.

[ref55] Zaki M. I.; Hasan M. A.; Al-Sagheer F. A.; Pasupulety L. In Situ FTIR Spectra of Pyridine Adsorbed on SiO_2_-Al_2_O_3_, TiO_2_, ZrO_2_ and CeO_2_: General Considerations for the Identification of Acid Sites on Surfaces of Finely Divided Metal Oxides. Colloids Surf., A 2001, 190 (3), 261–274. 10.1016/S0927-7757(01)00690-2.

[ref56] Harima Y.; Fujita T.; Kano Y.; Imae I.; Komaguchi K.; Ooyama Y.; Ohshita J. Lewis-Acid Sites of TiO_2_ Surface for Adsorption of Organic Dye Having Pyridyl Group as Anchoring Unit. J. Phys. Chem. C 2013, 117 (32), 16364–16370. 10.1021/jp405835y.

[ref57] Connor P. A.; Dobson K. D.; McQuillan A. J. Infrared Spectroscopy of the TiO_2_/Aqueous Solution Interface. Langmuir 1999, 15, 2402–2408. 10.1021/la980855d.

[ref58] León A.; Reuquen P.; Garín C.; Segura R.; Vargas P.; Zapata P.; Orihuela P. A. FTIR and Raman Characterization of TiO_2_ Nanoparticles Coated with Polyethylene Glycol as Carrier for 2-Methoxyestradiol. Appl. Sci. 2017, 7, 49 10.3390/app7010049.

[ref59] Panov V. P.; Kazarin L. A.; Dubrovin V. I.; Gusev V. I.; Kirsh Yu. É. Infrared spectra of atactic poly-4-vinylpyridine. J. Appl. Spectrosc. 1974, 21, 1504–1510. 10.1007/BF00604430.

[ref60] Castellà-Ventura M.; Akacem Y.; Kassab E. Vibrational Analysis of Pyridine Adsorption on the Brønsted Acid Sites of Zeolites Based on Density Functional Cluster Calculations. J. Phys. Chem. C 2008, 112, 19045–19054. 10.1021/jp8069354.

[ref61] Green I. X.; Buda C.; Zhang Z.; Neurock M.; Yates J. T. IR Spectroscopic Measurement of Diffusion Kinetics of Chemisorbed Pyridine through TiO_2_ Particles. J. Phys. Chem. C 2010, 114 (39), 16649–16659. 10.1021/jp1061489.

[ref62] Marwa B. M.; Bruno S.; Mongi B.; Van F. T.; Abdelmottaleb B. L. Modeling of Adsorption Isotherms of Dye N719 on Titanium Oxide Using the Grand Canonical Ensemble in Statistical Physics for Dye Sensitized Solar Cells. Sol. Energy 2016, 135, 177–187. 10.1016/j.solener.2016.05.015.

[ref63] Yoshida C.; Nakajima S.; Shoji Y.; Itoh E.; Momiyama K.; Kanomata K.; Hirose F. In Situ Observation of Structural Change in N719 Dye Molecule in Dye Sensitized Solar Cells with a Light Exposure and a Heat Treatment. J. Electrochem. Soc. 2012, 159 (11), H881 10.1149/2.053211jes.

[ref64] Rodrigues D. F. S. L.; Martins J.; Sauvage F.; Abreu C. M. R.; Coelho J. F. J.; Serra A. C.; Ivanou D.; Mendes A. Suppression of back electron recombination on the photoanode-electrolyte interface with poly(4-vinylbenzoic acid) and poly(4-vinylpyridine) co-adsorbents for stable and efficient dye-sensitized solar cells. Surf. Interfaces 2024, 44, 103627 10.1016/j.surfin.2023.103627.

[ref65] Martins J.; Emami S.; Ivanou D.; Mendes A. Ultralow Temperature Glass Frit Encapsulation for Stable Dye-Sensitized Solar Cells. ACS Appl. Energy Mater. 2022, 5 (11), 14185–14192. 10.1021/acsaem.2c02714. PMC9773422 36569782

[ref66] Capitão J.; Martins J.; Emami S.; Ivanou D.; Mendes A. Fully glass frit encapsulated dye-sensitized solar cells: Challenges for hermetical sealing of electrolyte injection holes. Sol. Energy 2023, 249, 476–484. 10.1016/j.solener.2022.12.001.

[ref67] Harikisun R.; Desilvestro H. Long-Term Stability of Dye Solar Cells. Sol. Energy 2011, 85 (6), 1179–1188. 10.1016/j.solener.2010.10.016.

[ref68] Moulay S. Molecular Iodine/Polymer Complexes. J. Polym. Eng. 2013, 33 (5), 389–443. 10.1515/polyeng-2012-0122.

[ref69] Luo J.; Lu Q.; Li Q.; Li Z.; Wang Y.; Wu X.; Li C.; Xie Y. Efficient Solar Cells Based on Porphyrin and Concerted Companion Dyes Featuring Benzo 12-Crown-4 for Suppressing Charge Recombination and Enhancing Dye Loading. ACS Appl. Mater. Interfaces 2023, 15 (35), 41569–41579. 10.1021/acsami.3c09187. 37608739

[ref70] Wang X.; Wang Y.; Zou J.; Luo J.; Li C.; Xie Y. Efficient Solar Cells Sensitized by Organic Concerted Companion Dyes Suitable for Indoor Lamps. ChemSusChem 2022, 15 (16), e202201116 10.1002/cssc.202201116. 35702052

