# Supplementary material for: Improved Interfacial Electron Dynamics with Block Poly(4-vinylpyridine)–Poly(styrene) Polymers for Efficient and Long-Lasting Dye-Sensitized Solar Cells
Source: ACS Appl Polym Mater. 2024 Jul 20;6(15):8939–49. doi: 10.1021/acsapm.4c01238 (PMC11320385; doi:10.1021/acsapm.4c01238)
Supplement: Supplementary file 1 — ap4c01238_si_001.pdf [file ap4c01238_si_001.pdf]

SUPPORTING INFORMATION

Improved interfacial electron dynamics with block poly(4-vinylpyridine)-poly(styrene) polymers for efficient and long-lasting dye-sensitized solar cells

Daniela F. S. L. Rodrigues [1,2], Carlos M. R. Abreu [1], Frédéric SAUVAGE[3], Jorge F. J. Coelho [1,4], Arménio C. Serra*[1], Dzmitry Ivanou*[2,5], Adélio Mendes [2,5]

[1]*University of Coimbra, CEMMPRE, ARISE, Department of Chemical Engineering*
Rua Sílvio Lima – Polo II, 3030-790 Coimbra, Portugal

[2]*LEPABE, Departamento de Engenharia Química, Faculdade de Engenharia, Universidade do Porto*
Rua Dr. Roberto Frias, 4200-465 Porto, Portugal

[3]*Laboratoire de Réactivité et Chimie des Solides, Université de Picardie Jules Verne (UPJV), CNRS UMR 7314, Hub de l'énergie, 15 rue Baudelocque, 80039 Amiens, France.*

[4]*IPN, Instituto Pedro Nunes, Associação para a Inovação e Desenvolvimento em Ciência e Tecnologia, Rua Pedro Nunes, 3030-199 Coimbra, Portugal*

[5]*ALiCE - Associate Laboratory in Chemical Engineering, Faculty of Engineering, University of Porto, Rua Dr. Roberto Frias, Porto 4200-465, Portugal*

*Corresponding authors: *E-mail address:* ivanou@fe.up.pt (D. Ivanou); armenio.serra@gmail.com (A. Serra)

Number of pages: 10

Number of figures: 5

Number of tables: 3

Number of schemes: 1

1. Synthesis of Poly(4-vinylpyridine)-*b*-poly(styrene) (P4VP-*b*-PSt) block copolymers

1.1. Reagents

4-vinyl pyridine (4VP) (Alfa Aesar, 96 %) was distilled before used, 2-(((dodecylthio)carbonothioyl)thio)propanoic acid (DoPAT) (Boron Molecular, > 97 %), styrene (St) (Acros organics, 99 %), N,N-dimethylformamide (DMF) (Fisher Scientific, > 99.5%), diethyl ether (JMGS, Lda, 99.88 %), Chloroform (JMGS, Lda, 99.98 %), deuterated chloroform ($CDCl_3$) (Eurisotop, 99.80 %), DMF for High-performance liquid chromatography (Fisher, 99.99 %), Lithium bromide (LiBr, Acros organics, 99+ %). AIBN (Fluka, 98 %) was recrystallized three times from ethanol before use.

2.1. Synthesis of P4VP-*b*-PSt block copolymers

First, the P4VP macroRAFT Agent was synthesized as described in elsewhere[1] to obtain a block homopolymer of P4VP. The obtained P4VP macroRAFT had an MW of 7.4k and a low *Đ* (≤ 1.3).

P4VP-b-PSt was synthesized by Reversible Addition-Fragmentation Chain Transfer (RAFT) polymerization using a P4VP macroRAFT as chain transfer agent (CTA) as shown in **Scheme S1.**

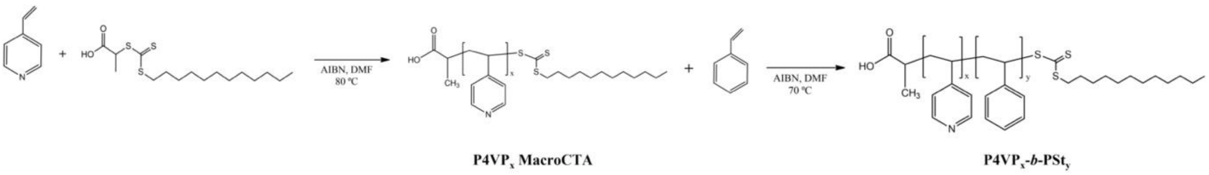

Scheme S1. Synthesis of P4VP-*b*-PSt block copolymer by RAFT polymerization initiated by AIBN.

The block copolymerization of P4VP-*b*-PSt was performed using the molar ratio: $[St]_0/[P4VP\ MacroCTA]_0/[AIBN]_0$ = 90/1/0.62.

Into a 50 mL Schlenk flask, 1.77 g of P4VP MacroCTA (0.24 mmol), St (2.5 mL, 21.53 mmol), AIBN (24.4 mg, 0.148 mmol) and DMF (7.5 mL) were added with a magnetic stirring bar. The Schlenk flask was closed and the mixture was degassed three times through freeze-evacuate-thaw cycles and placed under inert nitrogen atmosphere. The reactor was immersed in an oil bath at 70 ºC and stirred for 11 h. After this time, the flask was cooled down to room temperature and opened. The final reaction mixture was dissolved in $CHCl_3$ and precipitated in cold diethyl ether under stirring; the dissolution/precipitation process was repeated three

times. The polymer was dried at 40 ºC under vacuum oven until constant weight.

2. Characterization of the P4VP-*b*-PSt block copolymers produced

^{1}H nuclear magnetic resonance (NMR) spectra of P4VP-*b*-PSt block copolymers were used to characterize the copolymers. The ^{1}H NMR spectra of samples were recorded in $CDCl_3$ with TMS as an internal standard using a Bruker Avance III 400 MHz spectrometer with a 5-mm TIX triple resonance detection probe. The structure, purity, and conversion of copolymers were confirmed and determined using MestRenova software version 6.0.2–5475. ^{1}H-NMR spectra of P4VP MacroCTA and P4VP-*b*-PSt are presented in **Figure S1**.

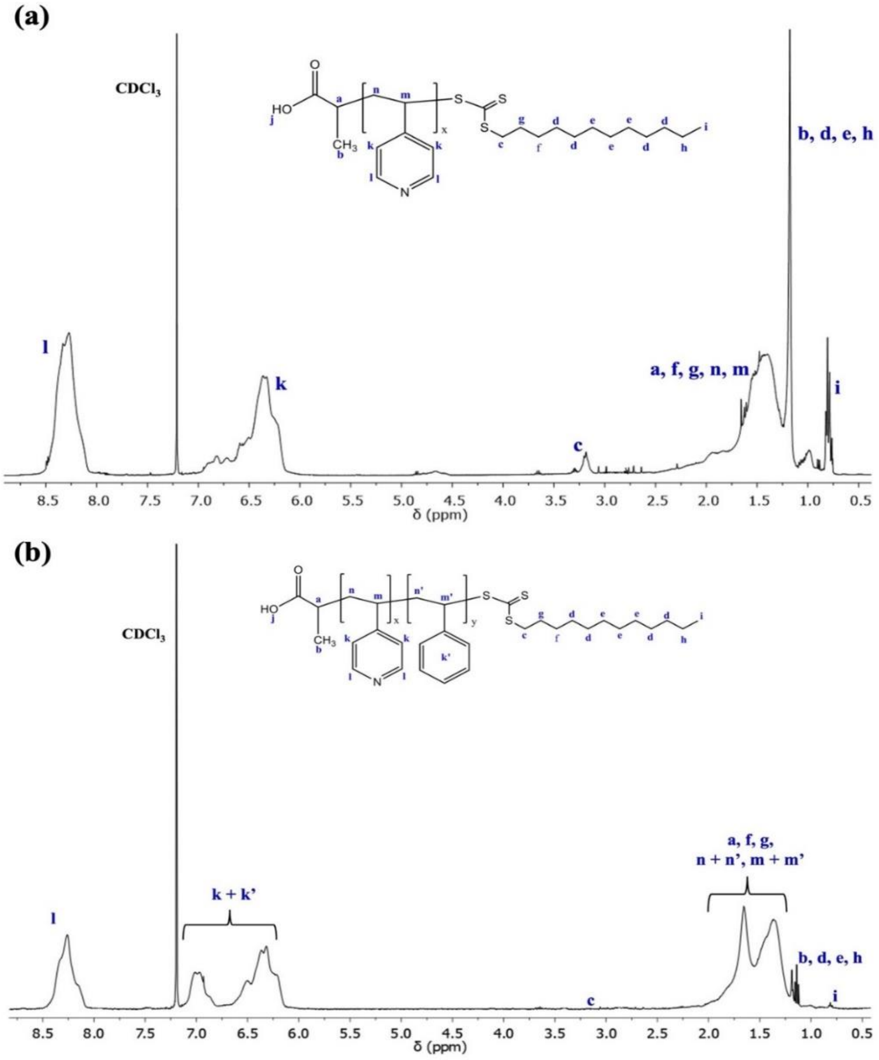

Figure S1. ^{1}H-NMR spectra of the P4VP MacroCTA **(a)** and P4VP-*b*-PSt block copolymer synthesized **(b)**.

The chromatographic parameters, such as molecular weight and dispersity ($Đ = M_w/M_n$), of P4VP MacroCTA and P4VP-*b*-PSt copolymers were determined using a size exclusion chromatography (SEC) from Viscotek (Viscotek TDAmax) equipped with a refractive index (RI), right-angle laser-light scattering (RALLS, Viscotek) and differential viscometer (DV), and low-angle laser light scattering (LALLS, Viscotek) detectors. The column set is comprised for a PLgel 5 µm guard column followed by one Viscotek T4000 column, one Viscotek D2000 column, and one Styragel column (5 µm). DMF with LiBr (0.03% w/w) was used as eluent with a flow rate of 1 mL min^{-1} at 60 ºC and it was previously filtered by a filter of 0.2 µm. The system was also equipped with an online degasser. The samples were filtered through a PTFE membrane with 0.2 µm pore before the injection (100 µL). The system was calibrated with five narrow poly (methyl methacrylate) (PMMA) standards (M_n of 1860, 4250, 9680, 20310, 50000). *Đ* and molecular weight (M_n^{SEC}) of synthesized polymers were determined from the conventional calibration using the OmniSEC software version 4.6.1.354.

Two block copolymers of P4VP-*b*-PSt were synthesized with molecular weights of 9780 g·mol^{-1} and 13740 g·mol^{-1} and a low dispersity ($Đ \leq 1.3$). The polymerization conditions and macromolecular characteristics are resumed in **Table S1**. **Figure S2** shows the molecular weight distribution curves of copolymers and P4VP MacroCTA obtained by SEC.

Table S1. RAFT polymerization and macromolecular characteristics of P4VP MacroCTA and P4VP-*b*-PSt copolymers.

Sample	CTA	St content (%)	$[M]_0/[CTA]_0/[I]_0$ [a]	Time (h)	S/M[b] (v/v)	T (ºC)	Conv. (%)	M_n (x 10^{-3}) Theoretical	^{1}H-NMR	SEC	*Đ*
$P4VP_{67}$	DoPAT	0	92/1/0.62	2.5	1/1	80	83.5	8.45	7.52 [c]	7.41	1.34
$P4VP_{67}$-*b*-PSt_{23}	$P4VP_{67}$	25	90/1/0.62	24.3	3/1	70	28.3	10.03	9.78 [d]	9.94	1.38
$P4VP_{67}$-*b*-PSt_{61}	$P4VP_{67}$	47	150/1/0.62	72.7	3/1	70	48.7	15.00	13.74 [d]	13.76	1.29

[a] M: monomer; CTA: chain transfer agent; I: initiator, AIBN.

[b] S/M: solvent/monomer ratio (v/v)

[c] M_n^{NMR} (Figure **S1**a) = $[(\int(l)/H_l)/\int(c)/H_c)]$ x M (4VP) + M (DoPAT)

[d] M_n^{NMR} (Figure **S1**b) = $[(\int(k')/H_{k'})/\int(l)/H_l) \times$ DP (P4VP MacroCTA] x M (St) + M (P4VP MacroRAFT), where DP (P4VP MacroRAFT) = $[(\int(l)/H_l)/\int(c)/H_c)$ of the **Figure S1a**.

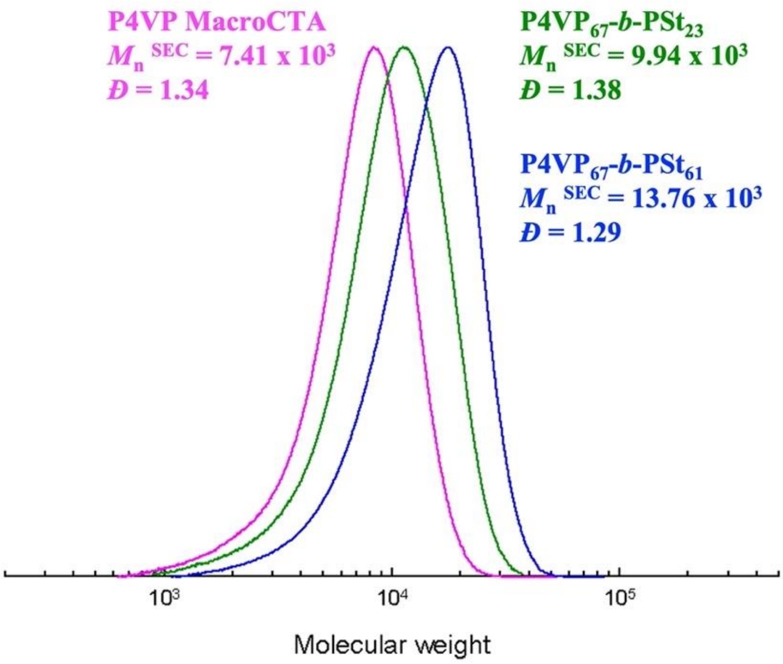

Figure S2. Normalized SEC traces of P4VP MacroCTA (pink line) synthesized by DoPAT-mediated RAFT polymerization and the extended P4VP$_{67}$-*b*-PSt$_{23}$ (green line) and P4VP$_{67}$-*b*-PSt$_{61}$ (blue line) block copolymers with different molecular weights after chain extension experiment by RAFT polymerization in DMF.

3. DSSCs Characterization

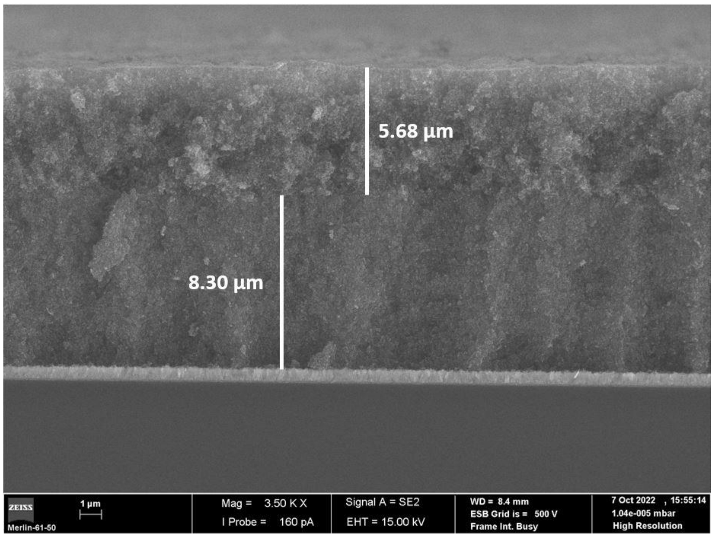

Figure S3. Cross-sectional SEM image of TiO_2 scaffold composed of 8.3 µm thick transparent TiO_2 and 5.7 µm thick active opaque layer.

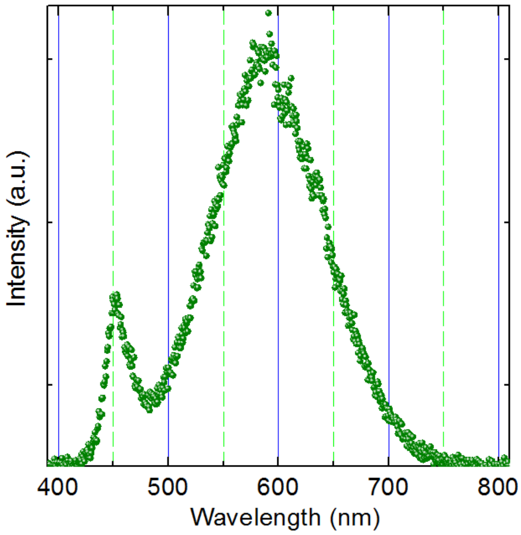

Figure S4. The emission spectrum of a white LED lamp (Color temperature 2700 K) used for *I-V* characterization of the DSSCs under artificial light.

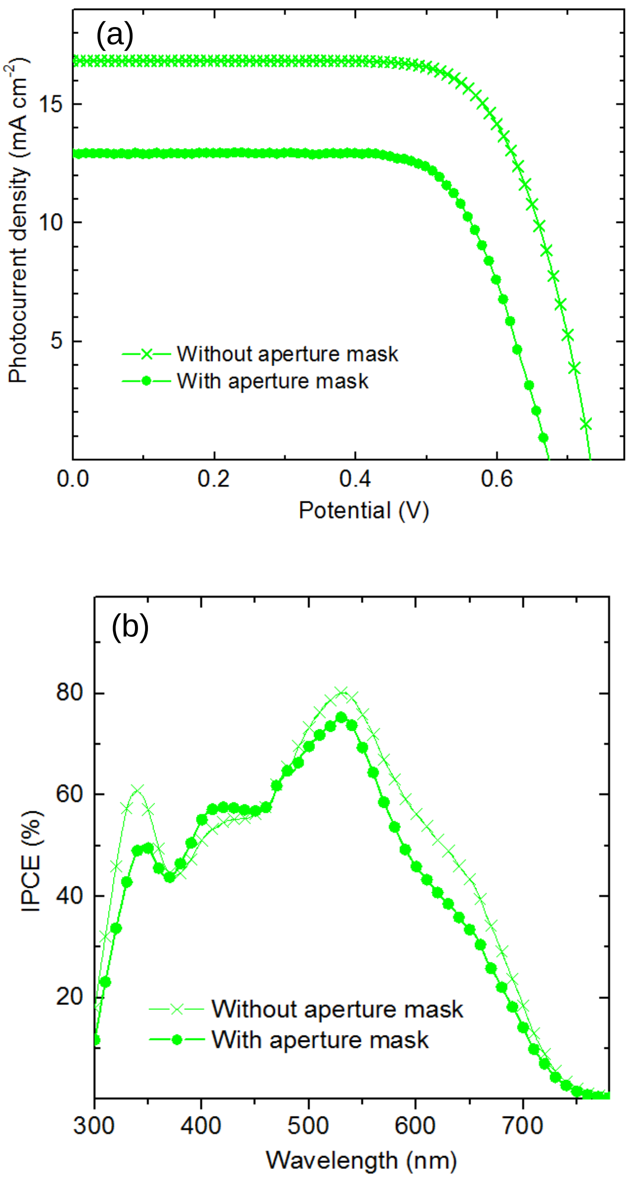

Figure S5. (a) Photocurrent under AM1.5G (100 mW cm^{-2}) without (crosses) and with (circles) aperture mask of DSSCs produced with the use of CDCA as co-adsorbent. (b) IPCE spectra of DSSCs with CDCA co-adsorment recorded without (crosses) and with (circles) aperture mask.

Table S2. Photovoltaic metrics in the beginning and at the end of the aging test. DSSCs devices with different co-adsorbents and EL-HPE electrolytes. The metrics were obtained without (-) and with (+) aperture masks.

Device	Aging time (h)	Mask	V_{OC} (V)	J_{SC} (mA cm^{-2})	FF	PCE (%)
P4VP	16	(-)	0.74±0.01	18.7±0.2	0.67±0.01	9.4±0.2
		(+)	0.71±0.01	13.9±0.2	0.73±0.01	7.4±0.2
	1144	(-)	0.71±0.01	11.5±0.2	0.47±0.01	3.8±0.2
		(+)	0.67±0.01	15.5±0.2	0.41±0.01	4.2±0.2
P4VP$_{67}$-*b*-PSt$_{23}$	16	(-)	0.77±0.01	18.0±0.2	0.65±0.01	9.1±0.2
		(+)	0.73±0.01	13.3±0.2	0.72±0.01	7.1±0.2
	1144	(-)	0.69±0.01	20.2±0.2	0.46±0.01	6.4±0.2
		(+)	0.65±0.01	14.9±0.2	0.60±0.01	5.8±0.2
P4VP$_{67}$-*b*-PSt$_{61}$	16	(-)	0.73±0.01	17.1±0.2	0.65±0.01	8.4±0.2
		(+)	0.68±0.01	11.2±0.2	0.74±0.01	5.8±0.2
	1144	(-)	0.71±0.01	20.9±0.2	0.48±0.01	7.1±0.2
		(+)	0.66±0.01	15.0±0.2	0.60±0.01	5.9±0.2

Table S3. Photovoltaic metrics in the beginning and at the end of the aging test. DSSCs devices with different co-adsorbents and EL-HSE electrolytes. The metrics were obtained without (-) and with (+) aperture masks.

Device	Aging time (h)	Mask	V_{OC} (V)	J_{SC} (mA cm^{-2})	FF	PCE (%)
P4VP	16	(-)	0.73±0.01	17.7±0.2	0.49±0.01	6.5±0.2
		(+)	0.70±0.01	13.3±0.2	0.59±0.01	5.6±0.2
	1371	(-)	0.80±0.01	12.7±0.2	0.58±0.01	5.9±0.2
		(+)	0.77±0.01	9.8±0.2	0.65±0.01	5.0±0.2
P4VP$_{67}$-*b*-PSt$_{23}$	16	(-)	0.73±0.01	15.4±0.2	0.50±0.01	5.8±0.2
		(+)	0.71±0.01	12.6±0.2	0.58±0.01	5.3±0.2
	1371	(-)	0.78±0.01	14.8±0.2	0.50±0.01	5.8±0.2
		(+)	0.75±0.01	11.6±0.2	0.59±0.01	5.1±0.2
P4VP$_{67}$-*b*-PSt$_{61}$	16	(-)	0.72±0.01	18.4±0.2	0.54±0.01	7.3±0.2
		(+)	0.69±0.01	14.5±0.2	0.64±0.01	6.5±0.2
	1371	(-)	0.73±0.01	17.4±0.2	0.58±0.01	7.3±0.2
		(+)	0.69±0.01	13.1±0.2	0.67±0.01	6.0±0.2

References

(1) Rodrigues, D. F. S. L.; Santos, F.; Abreu, C. M. R.; Coelho, J. F. J.; Serra, A. C.; Ivanou, D.; Mendes, A. Passivation of the TiO_2 Surface and Promotion of N719 Dye Anchoring with Poly(4-vinylpyridine) for Efficient and Stable Dye-Sensitized Solar Cells. *ACS Sustainable Chem. Eng.* **2021**, *9* (17), 5981–5990. DOI: 10.1021/acssuschemeng.1c00842
